# Elevated MSH2 MSH3 expression interferes with DNA metabolism *in vivo*

**DOI:** 10.1093/nar/gkad934

**Published:** 2023-11-01

**Authors:** Melisa Medina-Rivera, Samantha Phelps, Madhumita Sridharan, Jordan Becker, Natalie A Lamb, Charanya Kumar, Mark D Sutton, Anja Bielinsky, Lata Balakrishnan, Jennifer A Surtees

**Affiliations:** Department of Biochemistry, Jacobs School of Medicine and Biomedical Sciences, University at Buffalo, Buffalo NY, 14203, USA; Department of Biochemistry, Jacobs School of Medicine and Biomedical Sciences, University at Buffalo, Buffalo NY, 14203, USA; Department of Biology, Indiana University Purdue University Indianapolis, Indianapolis, IN, 46202, USA; Department of Biochemistry, Molecular Biology and Biophysics, University of Minnesota, Minneapolis, MN, 55455, USA; Department of Biochemistry, Jacobs School of Medicine and Biomedical Sciences, University at Buffalo, Buffalo NY, 14203, USA; Department of Biochemistry, Jacobs School of Medicine and Biomedical Sciences, University at Buffalo, Buffalo NY, 14203, USA; Department of Biochemistry, Jacobs School of Medicine and Biomedical Sciences, University at Buffalo, Buffalo NY, 14203, USA; Department of Biochemistry, Molecular Biology and Biophysics, University of Minnesota, Minneapolis, MN, 55455, USA; Department of Biology, Indiana University Purdue University Indianapolis, Indianapolis, IN, 46202, USA; Department of Biochemistry, Jacobs School of Medicine and Biomedical Sciences, University at Buffalo, Buffalo NY, 14203, USA

## Abstract

The Msh2–Msh3 mismatch repair (MMR) complex in *Saccharomyces cerevisiae* recognizes and directs repair of insertion/deletion loops (IDLs) up to ∼17 nucleotides. Msh2–Msh3 also recognizes and binds distinct looped and branched DNA structures with varying affinities, thereby contributing to genome stability outside post-replicative MMR through homologous recombination, double-strand break repair (DSBR) and the DNA damage response. In contrast, Msh2–Msh3 promotes genome instability through trinucleotide repeat (TNR) expansions, presumably by binding structures that form from single-stranded (ss) TNR sequences. We previously demonstrated that Msh2–Msh3 binding to 5′ ssDNA flap structures interfered with Rad27 (Fen1 in humans)-mediated Okazaki fragment maturation (OFM) *in vitro*. Here we demonstrate that elevated Msh2–Msh3 levels interfere with DNA replication and base excision repair *in vivo*. Elevated Msh2–Msh3 also induced a cell cycle arrest that was dependent on *RAD9* and *ELG1* and led to PCNA modification. These phenotypes also required Msh2–Msh3 ATPase activity and downstream MMR proteins, indicating an active mechanism that is not simply a result of Msh2–Msh3 DNA-binding activity. This study provides new mechanistic details regarding how excess Msh2–Msh3 can disrupt DNA replication and repair and highlights the role of Msh2–Msh3 protein abundance in Msh2–Msh3-mediated genomic instability.

## Graphical Abstract

**Graphical Abstract:**
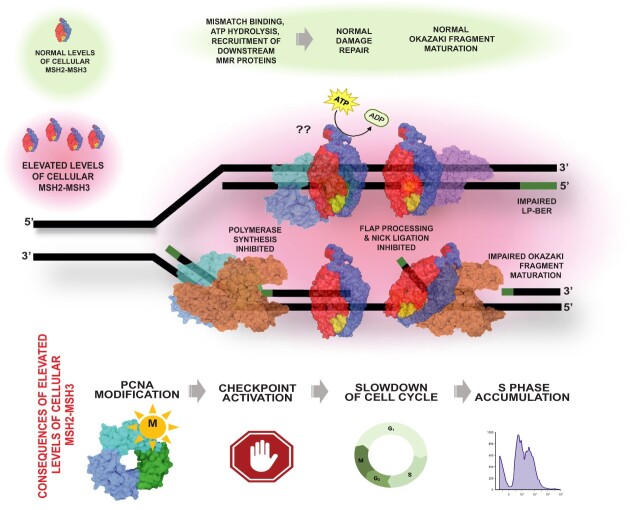

## Introduction

Mismatch repair (MMR) is a specialized DNA repair pathway known for its role in identifying and directing the correction of errors that evade the intrinsic fidelity mechanisms of the replication machinery, thereby increasing the fidelity of replication ∼100–1000-fold (1–4). In *Saccharomyces cerevisiae*, two heterodimeric MutS homolog (Msh) complexes initiate MMR with distinct but partially overlapping binding affinities (5,6). Msh2–Msh6 predominantly binds and directs the repair of single base mispairs (with the exception of C–C mismatches) and 1–2 nucleotide insertion-deletion loops (IDLs) (6–10). Msh2–Msh3 binds and directs repair of some mispairs, including A–A, C–C and T–G (10–12), as well as both short and longer IDLs of up to 17 nucleotides in length (13–16). Following mismatch recognition, MutL homologs (Mlh), Mlh1–Mlh3 and/or Mlh1–Pms1 (Pms2 in humans) are recruited by MSH–DNA complexes in an ATP-dependent manner. The endonuclease activity of Mlh homologs is directed to cleave the nascent strand distal to the mismatch, an activity that requires a Msh complex. Mlh1–Pms1 is also activated by proliferating cell nuclear antigen (PCNA) (17–19), while Mlh1–Mlh3 is not (20,21). Mlh1–Mlh2 lacks endonuclease activity and acts as an accessory factor (22,23). Subsequently, Exo1 and replicative DNA polymerase delta (Pol δ), or epsilon (Pol ϵ), are recruited to remove the error and resynthesize the DNA to restore the structure of the double helix (24–26).

Msh2–Msh3 is a structure-specific DNA-binding protein that binds a variety of different DNA intermediates, with a preference for substrates with double-strand (ds)/single-strand (ss) DNA junctions (13,14,27,28). This allows Msh2–Msh3 to initiate several pathways in DNA metabolism in addition to MMR. During 3′ non-homologous tail removal (3′NHTR), a step that occurs in a sub-class of DSBR (29–32), Msh2–Msh3 binds to ds/ssDNA junctions with 3′ ssDNA non-homologous tails to stabilize them and recruits the structure-specific endonuclease Rad1-Rad10/Saw1, thereby promoting cleavage of the unannealed tails, allowing repair to proceed via DNA synthesis (29,30,33). Msh2–Msh3 also promotes heteroduplex rejection, preventing recombination between homoleogous sequences (34,35). In this context, Msh2–Msh3 binds IDL structures, similar to MMR, but recruits Sgs1 to unwind the D-loop. (36,37). Given these known functions, it is not surprising that loss of Msh2–Msh3 is associated with an increase in genomic instabilities that contribute to hereditary and sporadic cancers in humans (38–50). At the same time, Msh2–Msh3 also promotes genome instability in structure-specific contexts. One notable example is trinucleotide repeat (TNR) sequences; Msh2–Msh3 binding promotes the expansion of (*CNG*) tracts and likely other repeat sequences that form secondary structures (51–57), including in *S. cerevisiae* (28,58,59). Similarly, Msh2–Msh3 binding to B-DNA/Z-DNA junctions promotes mutation (60). Thus, Msh2–Msh3 binding to non-canonical DNA structures can compromise genome stability. Overexpression of *MSH3* also results in a base-base mismatch repair deficiency that has been attributed to an imbalance of the relative protein ratios between Msh2–Msh3 and Msh2–Msh6 (61,62).

Msh2–Msh3 ATPase activity is required to promote genome stability through MMR, 3′ NHTR and heteroduplex rejection (63,64) and to promote genome instability through TNR expansions (65), although it is dispensable for DNA structure binding (14,64,66). Like MutS and Msh2–Msh6, Msh2–Msh3 contains two composite ATP-binding/hydrolysis sites with highly conserved Walker A and Walker B adenosine nucleotide-binding sites that are essential for ATP binding and hydrolysis, respectively (67–70). In *S. cerevisiae*, amino acid substitutions of the Walker A (G796 in Msh3), predicted to prevent ATP binding, abolished Msh2–Msh3-mediated MMR (64). Notably, Msh2–Msh3 binding to different DNA structures alters the kinetics of Msh2–Msh3 ATP binding, hydrolysis and nucleotide turnover, which impact downstream steps such as Msh2–Msh3 turnover and recruitment of partner proteins, promoting genome stability or instability (14,27,28,58,66,68,71,72).

We previously demonstrated that Msh2–Msh3 binds 5′ ssDNA flap structures, albeit with lower affinity than 3′ ssDNA flaps (14), to form a specific complex that interacts with the ss/dsDNA junction (28). 5′ ssDNA flaps are generated in at least two DNA metabolic pathways: Okazaki fragment maturation (OFM) during DNA replication and long-patch base excision repair (LP-BER). Polymerase (Pol) δ extends the initiator primer in OFM and primer upstream of the abasic site in LP-BER, eventually encountering the preceding DNA fragment. Pol δ proceeds with synthesis, displacing the 5′-end of the downstream segment, forming a single-stranded 5′ flap structure (73–75). This intermediate is cleaved by endonuclease Rad27 (Fen1 in mammals), leaving a nick that is sealed by DNA ligase Cdc9^LigI^ (DNA Ligase I (LigI) in humans). We demonstrated that Msh2–Msh3 competes with both Rad27^FEN1^ and Cdc9^LigI^ for binding to DNA substrates. This resulted in the inhibition of Rad27 ^FEN1^ endonuclease activity, ligation and a significant reduction of Okazaki fragment processing *in vitro* (28). Given that 5′ flap processing is essential for DNA metabolism *in vivo*, uncontrolled binding of Msh2–Msh3 to 5′ flap intermediates poses a potential risk for normal DNA metabolism.

Here we present evidence that elevated levels of Msh2–Msh3 interfere with DNA metabolism *in vivo* through multiple pathways, likely as a result of binding to non-canonical DNA structures. The cell responds to Msh2–Msh3’s interference with a checkpoint-like response. Msh3 is present at low levels in yeast, ∼4–10 times lower than Msh2 or Msh6 (76,77). Nonetheless, even low levels of Msh2–Msh3 overexpression increased sensitivity to the alkylating drug methyl methanesulfonate (MMS), which generates lesions typically repaired by base excision repair (BER). Msh2–Msh3 overexpression also induced defects in cell cycle progression that are likely a result of Okazaki fragment stress. Our results support a model in which elevated levels of Msh2–Msh3 interfere with normal DNA metabolism, not simply by binding DNA substrates but by engaging in aberrant signaling in an ATP binding-dependent manner. This work provides novel, mechanistic information demonstrating how Msh2–Msh3, known primarily to promote genome stability, can disrupt DNA replication and repair. Our data provide a more robust understanding of how the DNA metabolic pathways that generate a variety of DNA intermediates with double-strand/single-strand junctions are affected by elevated Msh2–Msh3 levels and provides a model for how the cell responds. Furthermore, they suggest that tight regulation of the Msh2–Msh3 expression levels is important *in vivo* to prevent interference with both DNA synthesis and the processing of a variety of DNA structures.

## Materials and methods

### Plasmids and yeast strains

To generate low-copy and high-copy plasmids expressing *MSH3*, a *Sac*II–*Pst*I fragment from pEAI215 (71), which includes DNA sequence from ∼1 kb upstream of *MSH3* to ∼ 500 bp downstream of *MSH3* from its endogenous chromosomal location, was ligated into pRS423 (78) digested with *Sac*II and *Pst*I to generate pSP1. This is a high copy 2μ plasmid with a *LEU2* marker. From this plasmid, a *Sac*II–*Sal*I fragment was excised, containing the entire *MSH3* sequence from pEAI215 and ligated into pRS424 (78) and pRS414 (79) digested with *Sac*II and *Sal*I to generate pSP15 and pSP18, respectively. Both carry a *TRP1* marker; pSP15 is a 2μ plasmid, while pSP18 is a single copy *ARS CEN* plasmid. Plasmids were transformed into a *msh3Δ* yeast strain background using the lithium acetate method (80).

Overexpression plasmids of *MSH2* (pMMR8) and *msh2G693D* (pEAE270) (Supplementary Table S1) have been described previously (13,14,81). Galactose-inducible overexpression plasmids of *MSH3* (pMMR20), *msh3Y925A* (pCK94 or pMME2), *msh3G796A* (pCK42), *MSH6* (pEAE218) and empty vector (pJAS104) were described previously (Supplementary Table S1) (13,14,33,64,81). We generated *msh3D870A* in pEAI218 (71) by site-directed mutagenesis. A *Bsu36I*–*MluI* fragment from this plasmid, containing the *msh3D870A*, was sub-cloned into pMMR20 (13), to generate a galactose-inducible *msh3D870A* overexpression plasmid (pMME3). *MSH3*, *msh3*, or empty vector plasmids (all carrying the *leu2D* nutritional marker) were co-transformed with the *MSH2* or *msh2* overexpression plasmid (*TRP1* marker) into various yeast strains using the lithium acetate method (80). For the His-PCNA (*POL30*) and His-pcna (*pol30*) mutant experiments, yb2062 (*His-POL30*), yb2063 (*His-pol30K164R*), yb2064 (*His-pol30K242R*), yb2066 (*His-pol30K164R/K242R)*(82) were made *trp1^-^* and *leu2^-^* by sequential marker swap by *hisG-URA3-hisG* pop-out with pNKY85 (targeting *LEU2*) and pNKY1009 (targeting *TRP1*) (83,84) to generate JSY4937-4945 (*His-POL30*), JSY5007-5012 (*His-pol30K164R*). JSY5013-14 (*His-pol30K242R*) and JSY5015-27 (*His-pol30K164R/K242R*). These strains were co-transformed with pMMR8 and pMMR20 or pJAS104.

All strains used in this study are described in Supplementary Table S2.

### Galactose inducible overexpression

Cultures of a *msh3Δ* (JSY1505 or JSY905) or *His-POL30*/*pol30* backgrounds carrying both pMMR8 (*MSH2*) or pMMR8-derived (*msh2G693D*) and pMMR20 (*MSH3*) or pMMR20-derived (*msh3* alleles) (13) were grown to mid-log phase in synthetic complete (SC) medium in the presence of 2% lactate and 2% glycerol as carbon sources. Protein expression was induced by the addition of 2% galactose for 17 h. Uninduced and induced cells were collected for flow cytometry, quantitative real-time PCR (qRT-PCR), and/or western blotting to analyze PCNA modification. Cells harvested for flow cytometry were washed with sterile deionized water and fixed in 70% ethanol at 4°C for a minimum of 1 h (up to 1 week) before flow cytometry analysis. Cells harvested for RNA extraction were washed with UltraPure™ DNase/RNase-Free Distilled Water (Invitrogen), harvested by centrifugation and resuspended in β-mercaptoethanol/Buffer RLT solution as described by RNeasy Mini Kit (QIAGEN) guidelines. Aliquots from each time point were snap-frozen in liquid nitrogen and stored at -80°C until ready for processing.

### RNA isolation and quantitative real-time PCR

Total RNA was isolated from cultured yeast cells using the QIAGEN RNeasy Mini Kit. As recommended by the manufacturer, residual DNA was removed by on-column DNase I digestion was carried out for 15 min. RNA concentration was determined via nanodrop. One μg of total RNA was reverse transcribed using a mix of oligo(dT) and random hexamer primers following the manufacturer's instructions of the iScript^TM^ cDNA synthesis kit (BioRad). Primers to detect endogenous transcript levels of *MSH2*, *MSH3*, *MSH6* and *PDA1* are described in Supplementary Table S3. The RT-PCR was performed at 95°C for 3 min, 40 cycles of amplification consisting of denaturation at 95°C for 15 s, annealing at 55°C for 30 s, extension at 72°C for 30 s, followed by melting curve analysis, using a CFX-95 Touch Multiplex instrument. Each RNA extraction was performed a minimum of three times and each qPCR experiment was performed in triplicate. Each PCR included a standard curve with genomic DNA, and the levels of target transcripts were normalized to that of the reference gene that encodes Pyruvate Dehydrogenase Alpha 1 (*PDA1*). The generation of specific PCR products was confirmed by gel electrophoresis. Experiments were done in the absence of reverse transcriptase and visualized by electrophoresis to determine lack of DNA contamination. The relative starting quantities (SQ) of mRNAs for *MSH* genes and *PDA1* were calculated from corresponding standard curves. Standard curves had an average *R^2^*> 0.98.

### Canavanine resistance assays

Mutation rates were measured at the *CAN1* locus as previously described (85,86). Briefly, strains were grown on SC–Trp–Leu plates until colonies reached 2 mm in size. The carbon source in the plates was 2% glucose or 2% galactose for *MSH3* uninduced or induced, respectively. Colonies were then suspended in 100 μl of 1× TE (10 mM Tris–HCl, pH 7.4; 1 mM EDTA) and diluted 1:10 000. Twenty μl of the undiluted colony suspension was plated on SC–Trp–Leu–Arg + Canavanine, and 100 μl of the 10^−4^ dilution was plated on SC–Trp–Leu–Arg. Both permissive and selective plates contained 2% glucose as a carbon source. The plates were incubated at 30°C until colonies reached ∼1–2 mm in size. Colonies were counted, and mutation rates and 95% confidence intervals were calculated through FluCalc fluctuation analysis software (87). Assays were performed on multiple independent isolates for each genotype on separate days.

### Methyl methanesulfonate survival assays

Cultures were grown to the mid-log phase in liquid SC–Trp media for assays performed with high and low-copy plasmids. Cells were diluted and plated on appropriate SC–Trp plates or SC–Trp plates containing 0.005, 0.01, 0.015, 0.0175 or 0.020% MMS. To avoid degradation of MMS and ensure consistent results, cells were plated within hours of pouring the plates. After incubation at 30°C for 4 days, percent survival was calculated as the ratio of the number of colonies that grew in the presence of MMS relative to the no MMS control. Assays were repeated a minimum of three times with at least two independent isolates.

For assays performed with overexpression strains, cultures were grown to the mid-log phase in SC media in the presence of lactate and glycerol as carbon sources. As described above, *MSH3* or *msh3* expression was induced by adding 2% galactose. Cells were diluted and plated into appropriate SC–Trp–Leu plates or SC–Trp–Leu plates containing 0.005% MMS.

### Cell cycle analysis by flow cytometry

After fixation in 70% ethanol, yeast cells were washed with sodium citrate/EDTA solution (50 mM sodium citrate and 1 mM EDTA [pH 8.0]) and treated with 0.06 mg RNase A (Invitrogen) at 50°C for 2 h. This was followed by adding 0.25 mg of Proteinase K (Sigma-Aldrich) and incubation at 50°C for 1–2 h. Cells were mixed with a sodium citrate/EDTA solution containing 1 μM SYTOX® Green. Stained DNA was then analyzed for chromosomal content using a BD Fortessa Flow Cytometer at an excitation wavelength of 488 nm. Data shown were analyzed using BD FACSDiva™ and FlowJo™ software.

### Detection of PCNA modification by western blot

Total protein extracts from yeast strains overexpressing Msh2–Msh3 were TCA precipitated and analyzed by Western blot with an anti-PCNA antibody as described previously (88,89). Blots were imaged with Bio-Rad Chemi-Doc Touch Imaging System. Linear changes in exposure were applied to entire blots. PCNA signal intensity was quantified using ImageJ software.

### DNA substrates

Oligonucleotides were synthesized by Integrated DNA Technologies (IDT, Coralville, IA) or Midland Certified Reagents Company (TX). For ATPase assays, the homoduplex (LS1/LS2) and +8 loop MMR (LS2/LS8) substrates sequences, assembly and purification were as described previously (14,66,71). Radiolabeled isotope was purchased from Perkin Elmer Life Sciences. Synthetic oligonucleotides were labeled with [γ ^32^P]-ATP and T4 polynucleotide kinase (New England Biolabs) at their 5′ end, as previously described (90). Following gel purification, substrate annealing was performed in a 1:2:4 ratio (labeled oligonucleotide: template: second oligonucleotide), as previously described (90). The oligonucleotides used in this study are listed in Supplementary Table S4.

### Protein purification

*S. cerevisiae* Msh2–Msh3 (66) and Pol δ (91) were expressed and purified as previously described.

### Pol δ strand extension assay

Two DNA substrates were used in the strand extension assays: the synthesis substrate and the strand displacement substrate. The synthesis substrate was created by annealing 5′ ^32^P-labeled 44mer to 110nt template sequence in a 1:2 ratio, respectively. The strand displacement substrate was formed by annealing a 5′ ^32^P-labeled 44 nucleotides upstream primer to a 110nt template containing a 60nt downstream primer in a 1:2:4 ratio. Five nM of the synthesis substrate was incubated with Pol δ (75 nM with the synthesis substrate and 150 nM for the strand displacement substrate) and increasing concentrations of Msh2–Msh3 (50, 100, 250 nM) for 30 min at 30°C. Reactions were performed in 20 μl volume in reaction buffer containing 50 mM Tris–HCl pH 8.0; 2 mM DTT, 0.25 mg/ml bovine serum albumin (BSA), 8 mM MgCl_2_, 1 mM ATP, 0.1 mM dNTPs, 25 mM NaCl. Reactions were terminated using 2× termination dye containing 90% formamide (v/v), 10 mM EDTA, with 0.01% bromophenol blue and xylene cyanole. Samples were then resolved on a 12% polyacrylamide gel containing 7 M urea. Products were analyzed by a PhosphorImager (Typhoon 9500) and quantified using ImageQuant version 5.2 (Molecular Dynamics). All experiments were done at least in triplicate. Representative gels are shown.

### ATPase assays

Hydrolysis of ATP was monitored using a coupled spectrophotometric assay as described previously (66,92). In this assay, the conversion of ATP to ADP and P_i_ is linked to the oxidation of NADH to NAD^+^ and is monitored as a decrease in absorbance at 340 nm. Assays were performed at 30°C and monitored using a Varian, Cary-50 Bio UV–vis spectrophotometer. The reactions contained 20 mM Tris-acetate (pH 7.5), 0.3 mM NADH, 5 mM PEP, 20 U/ml pyruvate kinase, 20 U/ml lactate dehydrogenase, 2 mM magnesium acetate, DNA (250 nM) and Msh2–Msh3 (50 nM) and up to 2.5 mM ATP. Msh2–Msh3 was pre-bound to DNA, followed by adding ATP in small increments. Approximately 80 data points were fit to a linear curve. The rate of ATP hydrolysis at each ATP concentration was calculated by multiplying the slope of the line by 159 (the change in absorbance of NADH per unit time) (92).

## Results

### Characterization of *MSH3* expression levels

Msh2–Msh3 binding interferes with the processing of 5′ ssDNA flap intermediates by Rad27^FEN1^ and Cdc9^LigI^*in vitro*, competing with Rad27^FEN1^ for binding the DNA intermediate (28). Because 5′ ssDNA flaps are generated in multiple DNA metabolic pathways, such as lagging strand synthesis, and long-patch BER, we were intrigued by the possibility that Msh2–Msh3 might interfere in these processes *in vivo*, leading to genome instability and replication stress. Notably, elevated levels of Msh2–Msh3 are a key driver of genome instability in eukaryotes *via* TNR expansions (93,94). Based on these results, we set out to test the hypothesis that elevated Msh2–Msh3 levels can drive genomic instability *via* 5′ flap DNA intermediates.

To address this hypothesis, we established a series of plasmids to express *MSH3* at different levels. First, we generated a single copy *ARS CEN* plasmid carrying *MSH3* under the control of its endogenous promoter (pSP18, *MSH3-LC*). Second, we constructed a high copy 2μ plasmid carrying *MSH3* under the control of its endogenous promoter (pSP15, *MSH3-HC*). Finally, *MSH2* and *MSH3* were co-overexpressed as described previously; *MSH2* (pMMR8) expression was under the control of the *ADC1* promoter, expressed constitutively and at elevated levels, and *MSH3* (pMMR20) was expressed under the control of a galactose-inducible *GAL-PGK* promoter (13,14).

**Figure 1.**
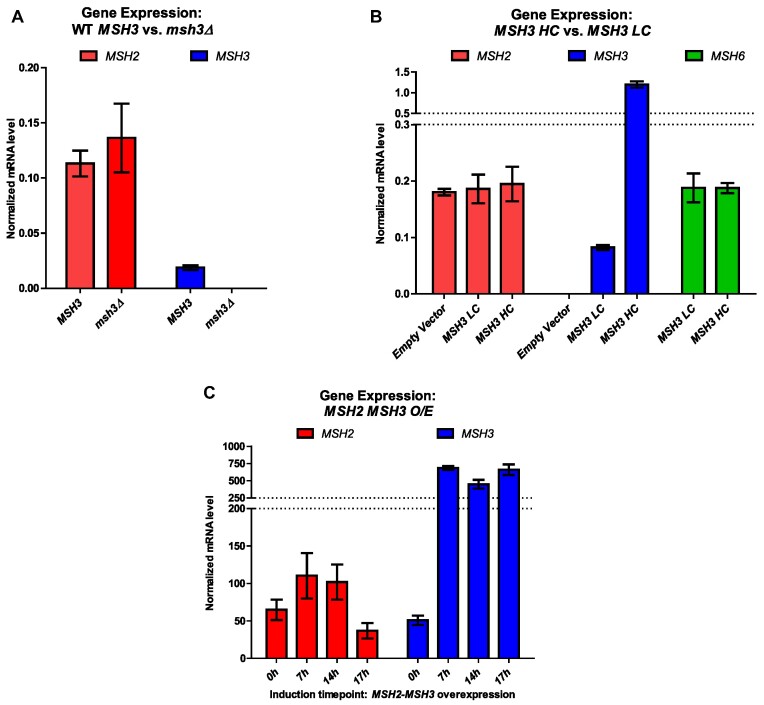
Expression levels of *MSH2*, *MSH3* and *MSH6* in different cellular contexts. Endogenous mRNA levels of *MSH2* (red), *MSH3* (blue) and *MSH6* (green) were measured using RT-qPCR. (**A**) RNA was isolated from *MSH3* or *msh3Δ* yeast cells at mid-log phase. (**B**) RNA was isolated at mid-log phase from *msh3Δ* strains carrying either an empty vector or either a low copy number (*ARS CEN;* LC) or a high copy number (*2 micron;* HC) plasmid, bearing *MSH3* under the control of the endogenous *MSH3* promoter. (**C**) RNA was isolated from strains co-overexpressing *MSH2*, under a constitutive promoter, and *MSH3*, under a galactose-inducible promoter. Culture aliquots were collected at indicated hours (h) after induction with galactose. All RNA levels were normalized to the reference gene *PDA1*. Data represents the mean of at least three independent experiments. Error bars represent SEM.

The endogenous Msh3 protein levels are not detectable by western blot (64). Instead, we measured expression levels by qRT-PCR, although we note that there is not necessarily a linear relationship between gene expression and protein levels. RNA was extracted from wild-type and *msh3Δ* cells. Consistent with low endogenous Msh3 protein levels, we observed endogenous *MSH3* mRNA levels to be six times lower than endogenous *MSH2* or *MSH6* expression levels (Figure 1A, B). The *MSH3* mRNA levels were four and sixty-four times higher than *MSH3* endogenous levels when expressed from *MSH3-LC* or *MSH3-HC* plasmids, respectively (Figure 1A, B). No significant differences in *MSH2* or *MSH6* expression levels were observed in the presence of *MSH3-LC* or *MSH3-HC*. (Figure 1B).

The *msh3Δ* strain was co-transformed with *MSH2* (pMMR8), and a second plasmid bearing either a galactose-inducible copy of *MSH3* (pMMR20) or an empty vector control derived from pMMR20 (pJAS104 [EV]). Prior to galactose induction, we observed an increase in *MSH3* mRNA of approximately ∼2300-fold over *MSH3* endogenous levels in these strains (Figure 1A, C), indicating read-through transcription in the absence of galactose. No *MSH3* mRNA was observed in the control *msh3Δ* strain co-expressing *MSH2* and the empty vector (pJAS104) (Supplementary Figure S1). After induction by galactose for 17 h, *MSH3* expression levels increased to ∼14-fold compared to pre-induction (0h) levels, ∼32 000 times higher than the endogenous levels of *MSH3* mRNA (Figure 1A, C). Using these constructs, we previously demonstrated detectable Msh3 or msh3 protein levels following induction, including *msh3* alleles tested here, although protein levels are still low (64).

Studies in human cell lines demonstrated a strong Msh2–Msh6-specific mutator phenotype when *MSH3* is overexpressed, presumably because excess Msh3 competes with Msh6 for interactions with Msh2 (61,62). To functionally test *MSH3* overexpression in yeast, we performed canavanine resistance assays, a Msh2–Msh6-specific mutator assay following galactose-induced overexpression of *MSH3* (Table 1). *MSH3* overexpression (*O/E*) in galactose increased the mutation rate ∼9-fold over the rate observed in *MSH3 O/E* grown in glucose, similar to the elevated mutation rate in *msh6Δ*, consistent with the presence of elevated Msh3 protein. Growth of *WT* strains in galactose in the absence of *MSH3 O/E* did not have this effect (Table 1). *MSH3-HC* also increased the mutation rate, albeit to a lesser extent (∼2-fold), while *MSH3-LC* had no effect (Table 1).

**Table 1.** Canavanine resistance mutation rates

Genotype	Carbon source	Rate of canavanine resistance [95% confidence intervals]	Change (relative to glucose)
*WT* ^#^ (n = 22)	Glucose	3.9 × 10^−7^ [2.5 × 10^−7^–5.5 × 10^−7^]	1
*WT^#^* (n = 22)	Galactose	2.6 × 10–7 [1.7 × 10^−7^–3.7 × 10^−7^]	0.7
WT + MSH3 O/E* (*n* = 66)	Glucose	7.9 × 10^−7^ [6.5 × 10^−7^–9.3 × 10^−7^]	1
WT + MSH3 O/E* (*n* = 121)	Galactose	7.2 × 10^−6^ [6.6 × 10^−6^–7.9 × 10^−6^]	9.1
**Genotype**	**Carbon source**	**Rate of canavanine resistance [95% confidence intervals]**	**Change (relative to empty vector)**
*WT*+ *HC^&^* (n = 77)	glucose	4.2 × 10^−7^ [3.3 × 10^−7^–5.1 × 10^−7^]	1
*WT*+ *MSH3-HC^%^* (*n* = 77)	glucose	8.0 × 10^−7^ [6.6 × 10^−7^–9.5 × 10^−7^]	1.9
*WT*+ *LC^$^*^)^ (n = 77)	glucose	4.3 × 10^−7^ [3.4 × 10^−7^–5.2 × 10^−7^]	1
*WT*+ *MSH3-LC*^+^ (*n* = 77)	glucose	4.3 × 10^−7^ [3.5 × 10^−7^–5.1 × 10^−7^]	1

#FY23; * JSY321-323; & JSY263; % JSY264; $ JSY265; + JSY266.

### Overexpression of *MSH2 MSH3* leads to MMS sensitivity

**Figure 2.**
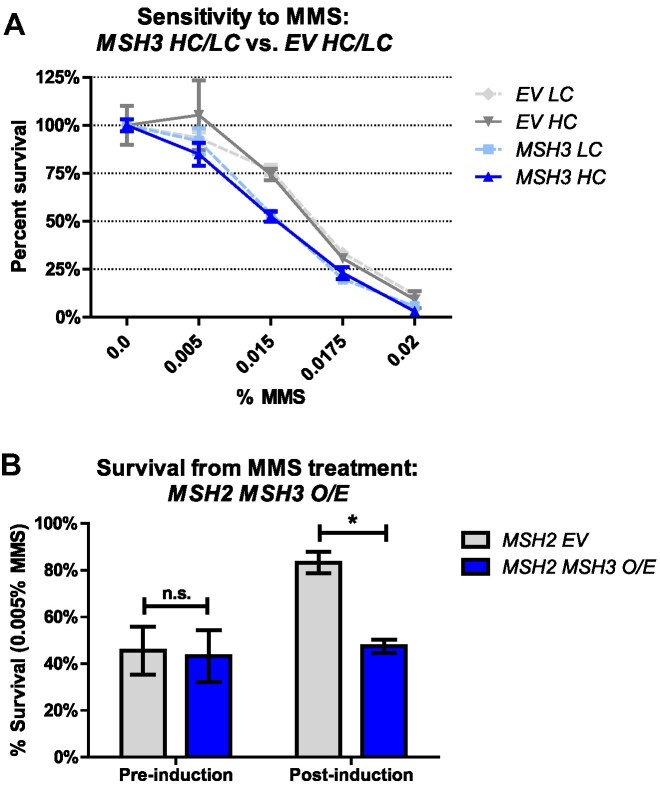
Elevated *MSH3* expression renders cells sensitive to methyl methanesulfonate (MMS). (**A**) MMS sensitivity of *msh3Δ* strains carrying either a low-copy (*MSH3 LC*, light shade) or high-copy number (*MSH3 HC*, dark shade) plasmid containing either empty vector (*EV*, gray lines) or *MSH3* (blue lines) were grown in increasing concentrations of MMS. Plotted graphs represent at least two independent experiments done in triplicate and with at least two independent transformants. Error bars were calculated by SEM; *P* values (two-way ANOVA) were calculated in Prism (*MSH3 LC* versus *EV LC P* < 0.0001; *MSH3 HC* versus *EV HC*, *P* = 0.0317). (**B**) MMS sensitivity of *msh3Δ* strains co-overexpressing *MSH2* and *MSH3*. Cultures were grown to mid-log phase and *MSH3* expression was induced by the addition of galactose. Time point 17 h after induction was collected, serial diluted and plated onto SC agar plates in the absence or presence of 0.005% MMS. Plotted data represents the results of at least three independent experiments. Error bars represent the SEM; *P* values (two-way ANOVA) were calculated in Prism (*P = 0.0297).

Given that Msh2–Msh3 interferes with 5′ flap processing *in vitro*, we tested whether elevated *MSH3* expression sensitized the cells to MMS, a monofunctional alkylation agent that methylates DNA at N^7^-deoxyguanine and N^3^-deoxyadenine (95), a lesion typically repaired by the BER pathway. *msh3Δ* yeast cells carrying *MSH3-LC* or *MSH3-HC* plasmids were grown to mid-log phase, serially diluted and grown on selective (SC-trp) plates in the absence or presence of MMS. At 0.015%, 0.0175% and 0.02% concentrations of MMS, cells carrying either *MSH3-LC* or *MSH3-HC* exhibited mild but significant sensitivity to MMS compared to empty vector controls (Figure 2A). When *MSH2* and *MSH3* were co-overexpressed (*MSH2 MSH3 O/E*), yeast cells exhibited significant sensitivity to 0.005% MMS compared to overexpression of *MSH2* alone (Figure 2B). With higher *MSH3* overexpression, cells were sensitive to much lower MMS concentrations, consistent with a correlation between Msh3 levels and MMS sensitivity. These results indicate that excess Msh2–Msh3 compromises LP-BER, suggesting interference with 5′ flap processing *in vivo. MSH2 MSH6* overexpression has also been shown to promote MMS sensitivity (96).

### Overexpression of *MSH2 MSH3* interferes with cell cycle progression

**Figure 3.**
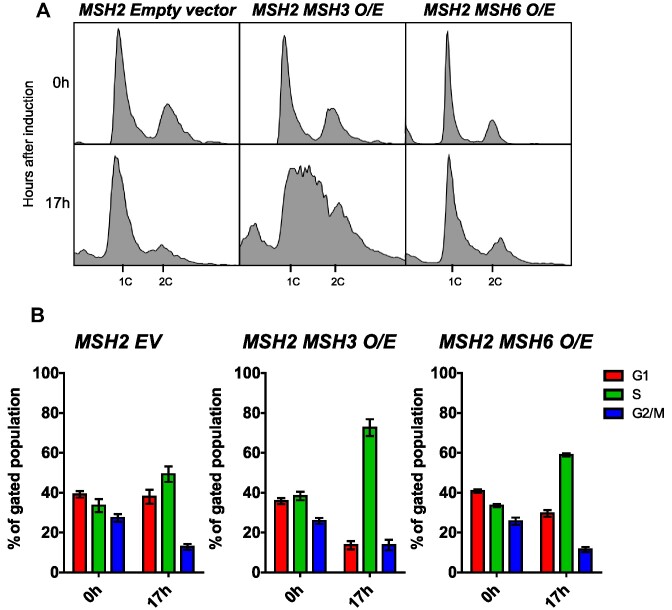
*MSH2 MSH3* overexpression induces a delay in cell cycle progression. *MSH2* and *MSH3* were overexpressed following galactose induction in a *msh3Δ* background. Aliquots were collected at 0 and 17 h after induction. (**A**) Histograms are shown of chromosomal content of asynchronous populations of *MSH2 + empty vector (EV), MSH2 + MSH3* and *MSH2 + MSH6* at several timepoints following addition of galactose. 1C indicates 1× DNA content; 2C indicates 2x DNA content. (**B**) Quantification of relative proportion of cells in different phases of the cell cycle for *MSH2 + EV*, *MSH2 + MSH3* or *MSH2 + MSH6*. The percentage of cells in G1 (1C), G2/M (2C) or S (between 1C and 2C) phases was determined using FlowJo software (see Supplementary Figure S2 for details). Plotted values correspond to data collected from at least three independent experiments from at least two independent isolates. Error bars represent SEM. Pairwise *t*-tests indicated significant differences in the number of cells in G1 and S phase between *MSH2 EV* and *MSH2 MSH3 O/E* (G1, *P*< 0.0001; S, *P* = 0.0026) and between *MSH2 MSH3 O/E* and *MSH2 MSH6 O/E* (G1, *P* = 0.0023; S, *P* = 0.0011).

**Figure 4.**
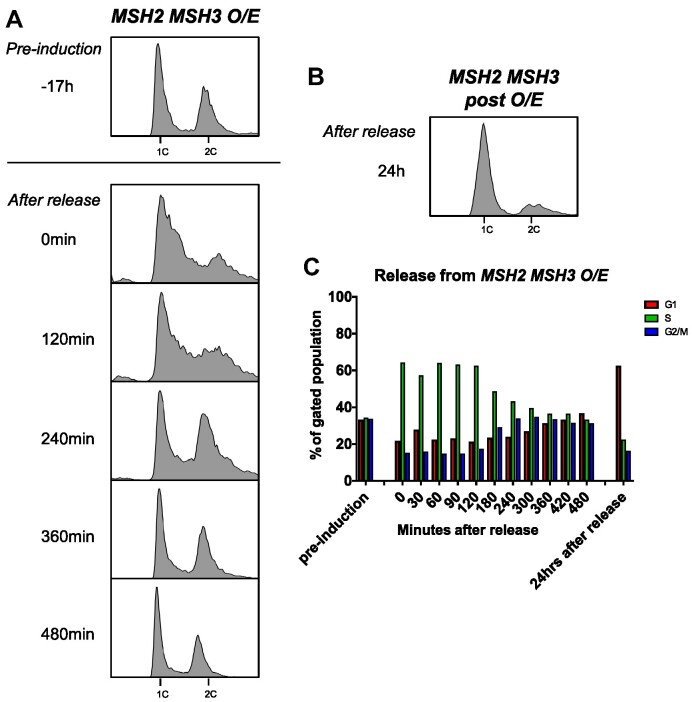
Recovery from *MSH2 MSH3* overexpression. *MSH2* and *MSH3* were overexpressed and induced as previously described. Cells were released from induction by transfer into glucose-containing media. Samples were collected and fixed for flow cytometry analysis (**A**) before induction (–17 h), after induction (0 min), after release into glucose every 30–60 min for 8 h, and (**B**) 24 h after release. Histograms show the distribution of chromosomal content at each time point. (**C**) Cell cycle profiles were quantified using FlowJo software (see Supplementary Figure S2).

Okazaki fragment maturation (OFM) also requires the processing of a displaced 5′ ssDNA flap; interference with OFM causes delays in cell cycle progression (82,89,97–100). Therefore, we assessed cell cycle progression by flow cytometry in cells overexpressing *MSH2* and *MSH3*. After *MSH2* and *MSH3* co-overexpression, *MSH2 MSH3 O/E* shown in Figure 1C, we observed substantial defects in cell cycle progression, which were not observed in the presence of the empty vector or *MSH2* and *MSH6* co-overexpression (Figure 3). Under these conditions, Msh3 protein is not visible in cleared lysates, whereas Msh6 is (data not shown;(7)), indicating high levels of Msh2–Msh6 upon overexpression compared with Msh2–Msh3. The cell cycle profile shifted dramatically, with an apparent accumulation of cells in the early S phase and a complete loss of defined G1 (1C) and G2/M (2C) populations. We quantified cell populations in G1, S or G2/M phases of the Cell Cycle function in FlowJo software (see Supplementary Figure S2). Upon *MSH2 MSH3* co-overexpression, we observed a substantial increase in the number of cells in the S phase with a concomitant decrease in cells in G1 and, to a lesser extent, in G2/M (Figure 3, Supplementary Figure S3). This accumulation of cells in the S phase is typically associated with slowed fork progression and/or DNA damage (101–103). These data suggest that overexpression of Msh2–Msh3 interferes with normal DNA metabolism, slowing down or inhibiting S phase. Notably, when the cells were released back into glucose, reversing the *MSH3* induction, they eventually resumed normal cell cycle progression (Figure 4). Notably, the cell cycle profile of cells co-overexpressing *MSH2 MSH3* resembled that of *rad27Δ* cells at elevated temperature (100), consistent with a model in which excess Msh2–Msh3 outcompetes Rad27^FEN1^*in vivo* and *MSH2 MSH3* overexpression makes the cells functionally *RAD27* null.

### *MSH3* overexpression enhances PCNA post-translational modification

**Figure 5.**
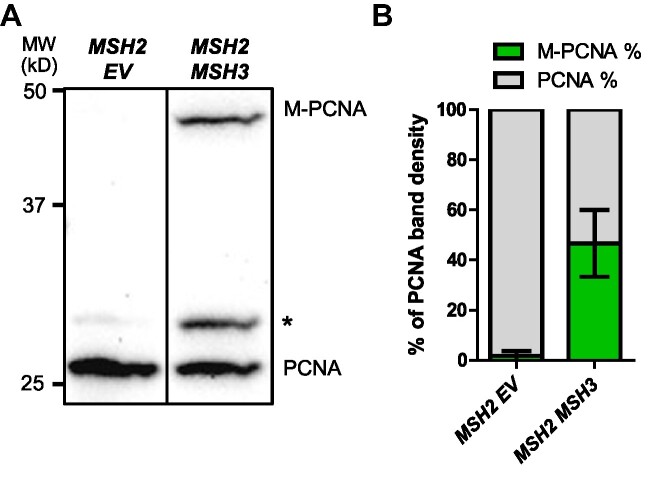
*MSH2 MSH3* overexpression induces post-translational modification of PCNA. *MSH2* and *MSH3* or empty vector (*EV*) were overexpressed in a *msh3Δ* background, as previously described. Following induction, TCA protein extracts were prepared, and the proteins were separated by 12% SDS-PAGE, transferred onto a membrane and then probed with anti-PCNA (P4D1), a gift from the Stillman lab. (**A**) Western blot for PCNA. Modified PCNA is marked as M-PCNA. The asterisk indicates non-specific bands. These data are from a single blot, but not adjacent lanes, as indicated by the vertical line. (**B**) PCNA western blot quantification, shown as relative band densities of modified versus unmodified PCNA in terms of the percentage of total PCNA signal (measured as the sum of unmodified and modified bands). A pairwise *t*-test was performed to test significance of the difference in the proportion of M-PCNA; *P*< 0.0001.

Interference with Okazaki fragment processing leads to cell cycle delays and DNA damage responses, signaling cascades that are partly mediated by post-translational modifications in PCNA (88,89,100). We examined whether *MSH2 MSH3* overexpression triggered PCNA modifications, which would support the hypothesis that a DNA damage response is activated. Following *MSH2 MSH3* overexpression, whole-protein TCA precipitation was performed, and the resulting cell extracts were analyzed by western blot with α-PCNA antibody (88,89). We observed two PCNA-specific bands, one consistent with the size of unmodified PCNA (∼29 kDa) (Figure 5A) and a PCNA band with lower mobility (∼49 kDa), consistent with a post-translationally modified form of the protein. This modified PCNA band was significantly increased following *MSH2 MSH3* overexpression compared to the empty vector (Figure 5B) and *MSH2 MSH6* overexpression (Supplementary Figure S4).

**Figure 6.**
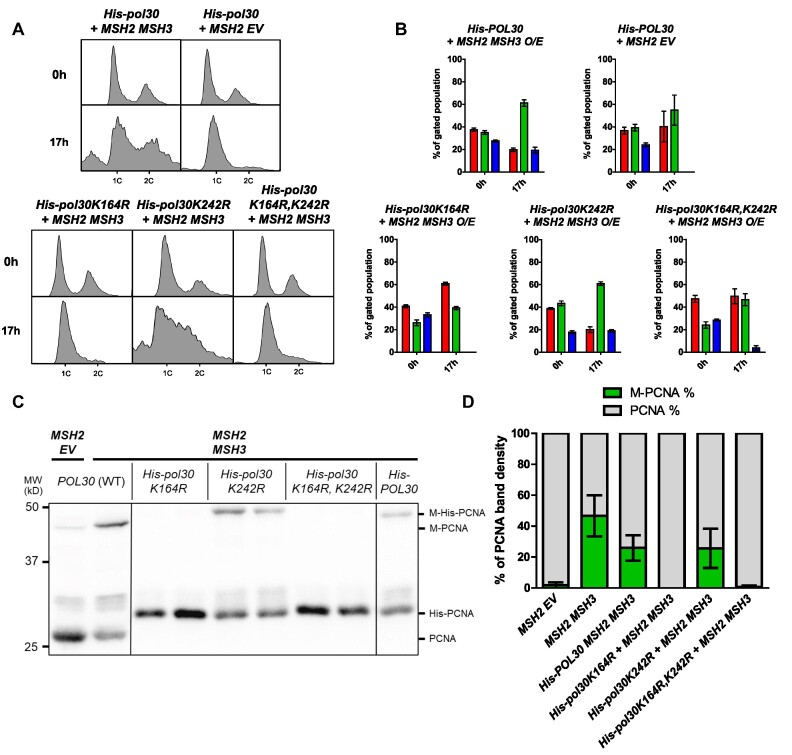
PCNA post-translational modification in the context of *MSH2 MSH3* overexpression occurs at the K164 residue. *MSH2* and *MSH3* or *MSH2* and empty vector were overexpressed following galactose induction in a *His-POL30, His-pol30K164R, His-pol30K242R*, or *His-pol30K164R + K242R*, background. Aliquots were collected and fixed for flow cytometry analysis before (0h) and after (17h) induction. After induction, cells were harvested for TCA extraction and anti-PCNA western blot. (**A**) Histograms are shown of chromosomal content of asynchronous populations before and after induction with galactose. Flow cytometry experiments were repeated at least three times, with at least two independent transformants. 1C indicates 1× DNA content; 2C indicates 2× DNA content. (**B**) Quantification of relative proportion of cells in different phases of the cell cycle. The percentage of cells in G1 (1C), G2/M (2C) or S (between 1C and 2C) phases was determined using BD FlowJo software (see Supplementary Figure S2 for details). Plotted values correspond to data collected from at least three independent experiments. Error bars represent SEM. Pairwise t-tests comparing the number of cells in G1 and S phase following *MSH2 MSH3 O/E* in *His-POL30* versus *His-pol30* alleles were performed. The counts were significantly different for *His-POL30* versus *His-pol30K164R* (*P*< 0.0001 for G1 and S phase cells) or *His-pol30K164R + K242R* (GI: *P* = 0.0098, S: *P*< 0.0001). In contrast, the cells counts were not significantly different between *His-POL30* and *His-pol30K242R* (G1: *P* = 0.8497; S: *P* = 0.8314). (**C**) PCNA western Blot. Note that the PCNA and M-PCNA of *his-POL30* strains migrates at a higher weight due to the His-tag. These data are from a single blot, but not adjacent lanes, as indicated by the vertical lines. (**D**) PCNA western Blot Quantification as described above. Pairwise *t*-tests indicated significant difference in the proportion of M-PCNA in *His-POL30* compared to *His-pol30K164R* or *His-pol30K164R + K242R* (p<0.0001) but not compared to *His-pol30-K242R* (*P* = 0.4152).

PCNA is differentially modified in response to distinct signaling pathways (88,104,105). To determine the residue at which PCNA is modified following *MSH2 MSH3* overexpression, we compared the modification of His-PCNA, His-pcnaK164R, His-pcnaK242R and His-pcnaK164R/K242R. Mutation of these highly conserved lysines to arginine renders them unmodifiable with either SUMO or ubiquitin. When *MSH2 MSH3* was overexpressed in the presence of *His-pol30-K164R* or *His-pol30-K164/RK242R*, modified PCNA was no longer detectable. The *His-pol30-K242R* exhibited modification levels similar to His-PCNA (Figure 6C). These results indicated that the *MSH2 MSH3* overexpression-dependent post-translational modification of PCNA occurs at K164 PCNA and not at K242.

**Figure 7.**
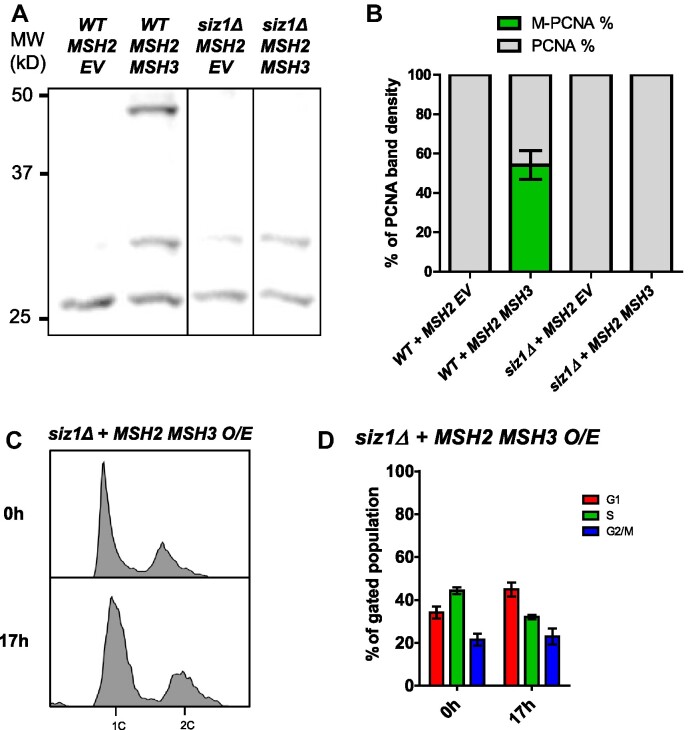
*MSH2 MSH3* overexpression-induced cell cycle defects are dependent on *SIZ1*. *MSH2* and *MSH3* were overexpressed in a *siz1Δ* background, as previously described. Samples were collected for flow cytometry analysis before and after induction, and cells were harvested for western blot after induction. (**A**) Western blot for PCNA. Modified PCNA is marked as M-PCNA. These data are from a single blot, but not adjacent lanes, as indicated by the vertical lines. (**B**) PCNA western blot quantification as previously described. The proportion of M-PCNA is significantly different in the *SIZ1* versus *siz1Δ* backgrounds (*P*< 0.0001) determined by a pairwise *t*-test. (**C**) Flow cytometry analysis before and after induction. (**D**) Quantification of cell cycle analysis based on flow cytometry data. Pairwise t-tests indicated significant difference in cell counts in G1 (*P*< 0.0001) and S (*P* = 0.0026).

We analyzed TCA-precipitated His-PCNA by western blot, using an α-ubiquitin (α-Ub) antibody (Supplementary Figure S5A) and were unable to detect the *MSH2 MHS3* overexpression-dependent modified PCNA band. In contrast, the *MSH2 MSH3* overexpression-dependent PCNA modification exhibited the same mobility as the PCNA modification induced by high MMS levels (Supplementary Figure S5B), which are known to promote PCNA sumoylation (105,106). Finally, the PCNA modification was abrogated in the absence of *SIZ1* (Figure 7A, B), which encodes the SUMO E3 ligase responsible for K164 sumoylation (107). Notably, the *MSH2 MSH3* overexpression-dependent cell cycle phenotype was also dependent on *SIZ1*. There was no S phase accumulation following *MSH2 MSH3* overexpression in *siz1Δ* (Figure 7C, D, Supplementary Figure S6).

The cell cycle analysis of these strains indicated that K164 is also required for the *MSH2 MSH3* overexpression-dependent accumulation of cells in S phase, while K242 was not essential. We note that the His-PCNA strain had a distinct cell cycle profile, with fewer cells in G2/M. Nonetheless, the accumulation of cells in the S phase remained clearly observable following *MSH2 MSH3* overexpression in this *HIS-POL30* background (Figures 6A, B).

### The *MSH2 MSH3* overexpression phenoype is *RAD9*-dependent

**Figure 8.**
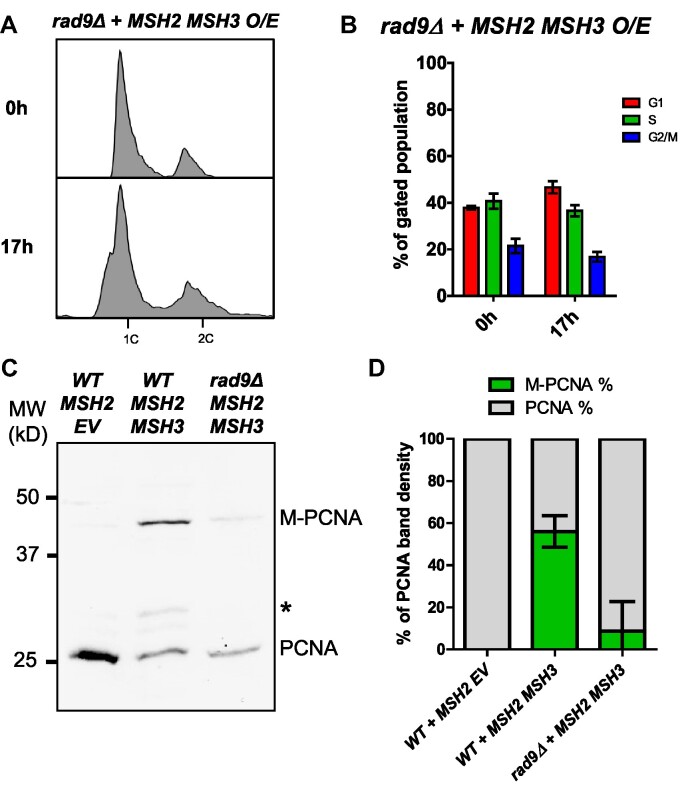
*MSH2 MSH3* overexpression-induced cell cycle defects are dependent on *RAD9*. *MSH2* and *MSH3* were overexpressed in a *rad9Δ* background, as previously described. Samples were collected for flow cytometry analysis before and after induction. (**A**) Flow cytometry analysis before and after induction. (**B**) Quantification of cell cycle analysis based on flow cytometry data. Pairwise *t*-tests indicated significant difference in cell counts in G1 and S phases (*P*< 0.0001) in *RAD9* versus *rad9Δ*. (**C**) Western blot for PCNA. Modified PCNA is marked as M-PCNA. (**D**) PCNA western blot quantification as previously described. The proportion of M-PCNA is significantly different in the *RAD9* versus *rad9Δ* backgrounds (*P*< 0.0001) determined by a pairwise *t*-test.

*MSH2 MSH3* overexpression leads to cell cycle delays, from which the cells recover, and post-translational modification of PCNA, all suggestive of activation of a cell cycle checkpoint response. Rad9, a cell cycle checkpoint protein involved in DNA damage signaling, is activated in response to impaired Okazaki fragment processing (89,108). We, therefore, overexpressed *MSH2 MSH3* in a *rad9Δ*. Deletion of *RAD9* was sufficient to suppress the cell cycle disruption caused by *MSH2 MSH3* overexpression (Figure 8A, B). The proportion of PCNA modified following *MSH2* MSH3 overexpression was also reduced in a *rad9Δ* background (Figure 8C, D), although there was some residual modification, ∼25% of what was observed in *RAD9* cells. These results indicated that a Rad9-mediated cell cycle checkpoint response becomes activated in the presence of excess Msh2–Msh3, leading to cell cycle arrest and PCNA modification, potentially as a result of Msh2–Msh3 interference with OFM.

### *ELG1* is required for PCNA modification and cell cycle defects caused by *MSH2 MSH3* overexpression

**Figure 9.**
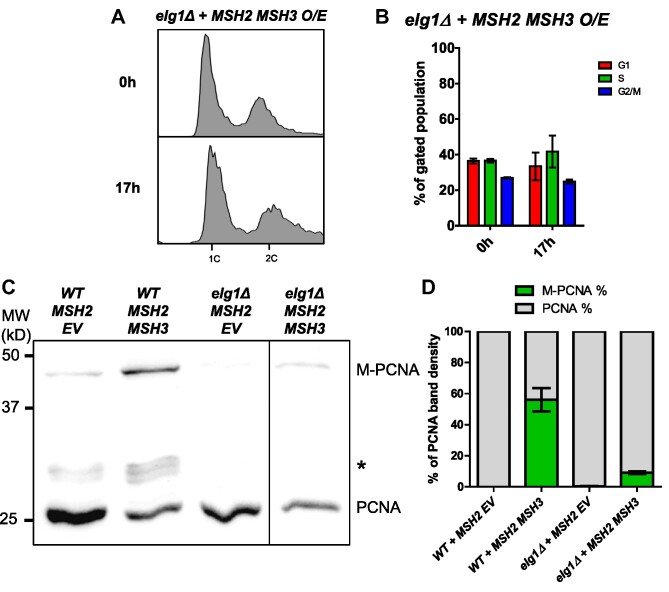
*MSH2 MSH3* overexpression-induced cell cycle defects are dependent on *ELG1*. *MSH2* and *MSH3* were overexpressed in an *elg1Δ* background, as previously described. Samples were collected for flow cytometry analysis before and after induction. (**A**) Flow cytometry analysis before and after induction. (**B**) Quantification of cell cycle analysis based on flow cytometry data. Pairwise *t*-tests indicated significant difference in cell counts in G1 (*P* = 0.0005) and S (*P* = 0.0022) in *ELG1* versus *elg1Δ*. (**C**) Western blot for PCNA. Modified PCNA is marked as M-PCNA. These data are from a single blot, but not adjacent lanes, as indicated by the vertical line. (**D**) PCNA western blot quantification as previously described. The proportion of M-PCNA is significantly different in the *ELG1* versus *elg1Δ* backgrounds (*P* = 0.0003) determined by a pairwise t-test.

During lagging strand synthesis, PCNA must be loaded onto DNA at each Okazaki fragment by Replication Factor C (RFC), while PCNA unloading is carried out by a related complex in which Elg1 replaces the primary subunit in RFC, known as the Elg1-Replication Factor C-like Complex (Elg1-RLC) (109). Elg1 unloads both unmodified and SUMOylated PCNA but preferentially binds to SUMOylated PCNA (110). Elg1 unloading of PCNA at each Okazaki fragment is dependent on successful processing and ligation of the Okazaki fragment (111). Therefore, we decided to test the effects of *MSH2 MSH3* overexpression in an *elg1Δ* background. We found that the *MSH2 MSH3* overexpression-dependent cell cycle defect was abrogated in *elg1Δ* (Figure 9, Supplementary Figure S6). We also observed reduced modification of PCNA (Figure 9). The requirement of *ELG1* for the cell cycle defect is consistent with the hypothesis that *MSH2 MSH3* overexpression interferes with Okazaki fragment processing *in vivo*.

### *msh3* alleles that disrupt ATP-binding and hydrolysis activities suppress disruption of cell cycle progression and PCNA post-translational modification

One possibility was that *in vivo*, excess Msh2–Msh3 binding to 5′ ssDNA flaps sterically interfered with Okazaki fragment maturation. Alternatively, Msh2–Msh3 bound to 5′ ssDNA flaps could promote aberrant Msh2–Msh3 activity that contributed to a signaling cascade leading to genome instability, similar to what is thought to occur in the presence of TNR structures (65,93). To distinguish between these possibilities, we tested the ability of ATPase-deficient *msh2* and *msh3* mutants to affect cell cycle progression. ATP is essential for the function and regulation of Msh2–Msh3 but is not required for DNA binding (13,14,63,64,66). Therefore, if Msh2–Msh3 simply binds to DNA structures and blocks Rad27^FEN1^, ATP binding, and/or hydrolysis should be dispensable for inducing the cell cycle defect. In fact, defects in these activities might even exacerbate the phenotype, as ATP hydrolysis contributes to Msh2–Msh3 turnover on the DNA (14,66). Alternatively, functional ATPase activity may be required to observe this effect, e.g. to recruit downstream proteins, as is the case for promoting TNR expansions (65,93).

**Figure 10.**
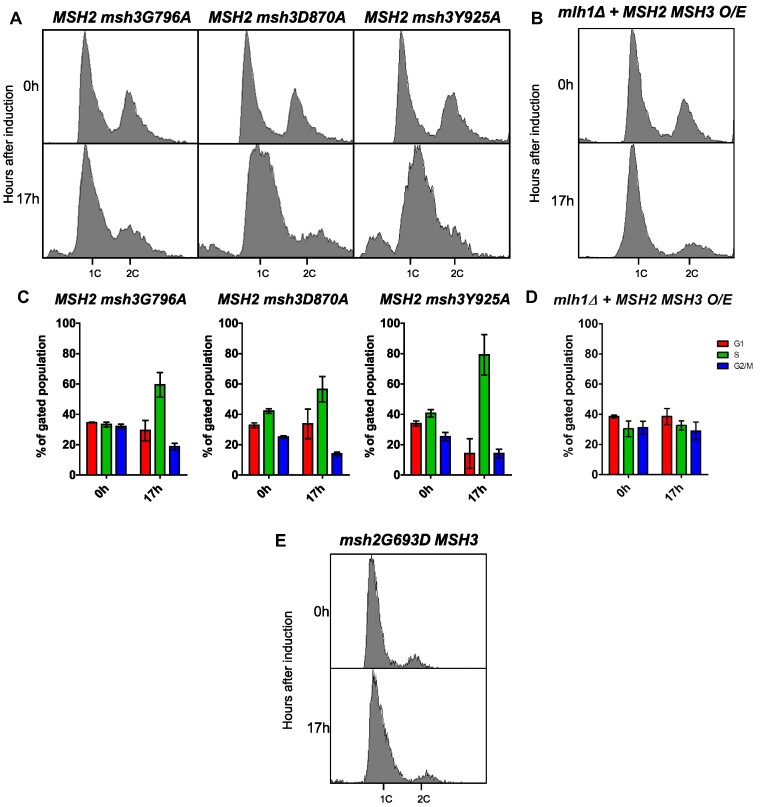
*MSH2 MSH3* overexpression-mediated cell cycle defect depends on Msh3 ATP binding and hydrolysis. *MSH2* (**A–D**) or *msh2G693D* (**E**) was co-overexpressed with *MSH3* (B, D) or *msh3* alleles (A, C) in a *msh3Δ* (A, C) or *mlh1Δ* (B, D) background. *MSH2* was constitutively overexpressed; for all others, expression was induced with galactose. Aliquots were collected at 0 and 17 h after induction. Harvested cells were fixed, stained and processed by flow cytometry. Histograms of the asynchronous population are shown. Pairwise *t*-tests were performed to determine whether observed differences in G1 and S cell counts were significantly different. We observed significant differences between *MSH2 MSH3* and *MSH2 msh3G796A* (G1: *P* = 0.0105; S: *P* = 0.0351) or *MSH2 msh2D870A* (G1: *P*< 0.0001; S *P* = 0.0007), but not *MSH2 msh3Y925A* (G1; *P* = 0.9298; S: *P* = 0.5414). We also observed significant differences between *MLH1* and *mlh1Δ* backgrounds (G1 and S, *P*< 0.0001).

To test these possibilities, we co-overexpressed *MSH2* and either *msh3G796A* or *msh3D870A* (64,93). These mutations disrupt the highly conserved Walker A motif (*msh3G796*), which mediates ATP binding, or the Walker B motif (*msh3D870*), which mediates ATP hydrolysis (64,68). Notably, the cell cycle defect was significantly less pronounced when either *msh3* allele was overexpressed compared to the overexpression of wild-type *MSH3* (Figure 10A, C). This suggests that full induction of the cell cycle defect requires Msh3 ATP binding and hydrolysis. Disruption of the Msh2 Walker A motif (*msh2G693D MSH3*) also suppressed the cell cycle defects when overexpressed (Figure 10E), indicating that ATP binding to both Msh2 and Msh3 is required for Msh2–Msh3 to disrupt cell cycle progression.

We overexpressed *MSH2 msh3Y925A*, a separation-of-function allele that is defective in MMR but functional in 3′NHTR (64). Based on the human Msh2–Msh3 crystal structure, Y925 is predicted to regulate nucleotide occupancy of the nucleotide-binding pocket by pushing a conserved phenylalanine (F940) into the nucleotide-binding pocket (68). *In vitro*, Msh2-msh3Y925A retains ATP hydrolysis activity, but the kinetics of hydrolysis are significantly altered, indicating a defect in the regulation of ATP binding, ATP hydrolysis, and/or nucleotide turnover (Supplementary Figure S7). When overexpressed, the *msh3Y925A* allele (Supplementary Figure S4) conferred cell cycle defects indistinguishable from the wild-type *MSH3* overexpression profile (Figure 10A, C). This is consistent with the hypothesis that ATP binding/hydrolysis by Msh2–Msh3 is required to impose cell cycle defects. However, regulation of this activity is less important, as observed for 3′ NHTR (64).

**Figure 11.**
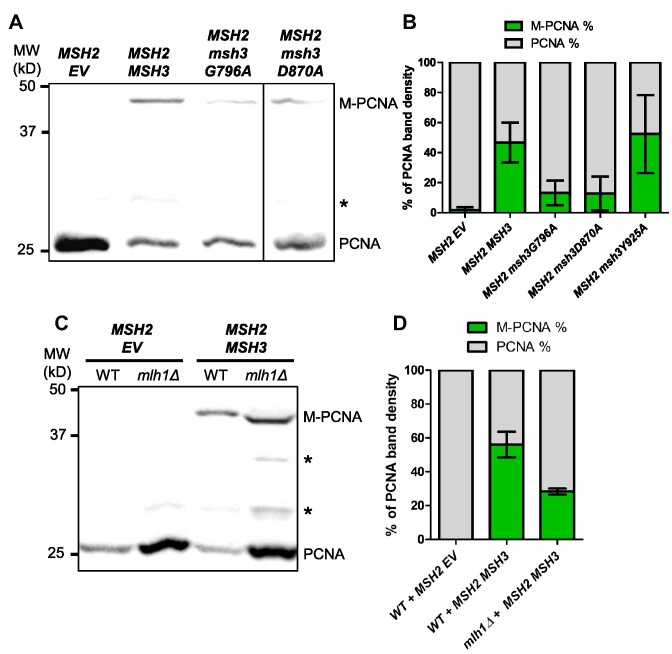
*MSH2 MSH3* overexpression induces PCNA post-translational modification in an ATP binding-and hydrolysis-dependent manner. *MSH2* and *MSH3* or *msh3* alleles were overexpressed in a *msh3Δ* (**A**) or *mlh1Δ* (**C**) background, as previously described. Western blot for PCNA. Modified PCNA is marked as M-PCNA. The data in (A) are from a single blot, but not adjacent lanes, as indicated by the vertical line. (**B, D**) PCNA western blot quantification as previously described. Pairwise *t*-tests were performed to determine whether the differences in the proportion of M-PCNA were significant. We observed significant differences between *MSH2 MSH3* and *MSH2 msh3G796A* or *MSH2 msh2D870A* (*P*< 0.0001), but not *MSH2 msh3Y925A* (*P* = 0.6901). We also observed significant differences between *MLH1* and *mlh1Δ* backgrounds (*P* = 0.0332).

We also analyzed the effects of these *msh3* alleles on PCNA post-translational modification. When either the Walker A (*msh3G796A*) or Walker B (*msh3D870A*) motif was disrupted, post-translational modification of PCNA in response to *MSH2 msh3* co-overexpression was decreased to a similar extent (Figure 11A,B). Co-overexpression of *MSH2 msh3Y925A* promoted PCNA modification (Supplementary Figure S4) at a level similar to wild-type, indicating that misregulated ATPase activity is sufficient for this effect. These results correlated with the intermediate effects of the Walker A and Walker B mutations and the wild-type effect of *MSH2 msh3Y925A* overexpression on the cell cycle progression phenotype (Figure 10C). *MSH2 MSH6* overexpression did not induce enhanced PCNA modification (Supplementary Figure S4).

These data indicate that an active Msh2–Msh3-mediated pathway alters cell-cycle progression and induces post-translational modification of PCNA that might indicate replication stress and/or activation of a DNA damage response with Msh2–Msh3 levels are elevated.

### Downstream steps in MMR are required for cell cycle progression defects when *MSH3* is overexpressed

The observation that Msh2–Msh3 ATP binding and hydrolysis activities are required to observe defects in cell cycle progression indicated that downstream steps in a Msh2–Msh3-mediated pathway might also be required to observe this phenotype. In MMR, Msh2–Msh3 DNA-binding leads to the recruitment of Mlh complexes and activation of their latent endonuclease activity (20,21,112–116). We tested whether *MLH1* is required for the cell cycle defect when *MSH2* and *MSH3* are co-overexpressed. We created a *mlh1Δ* strain, effectively inhibiting any downstream MMR activity by eliminating all three Mlh complexes: Mlh1–Pms1, Mlh1–Mlh2 and Mlh1–Mlh3. The *MSH2 MSH3* overexpression-dependent cell cycle defect was eliminated in the *mlh1Δ* background (Figure 10B,D; Supplementary Figure S6). These results indicate that one or more Mlh complexes contribute to Msh2–Msh3-mediated replication stress that disrupts the cell cycle. Notably, we still observed enhanced PCNA modification under these conditions (Figure 11C, D), indicating the elevated Msh2–Msh3 is sufficient for this effect.

### Msh2-Msh3 modulates DNA polymerase δ synthesis activity *in vitro*

**Figure 12.**
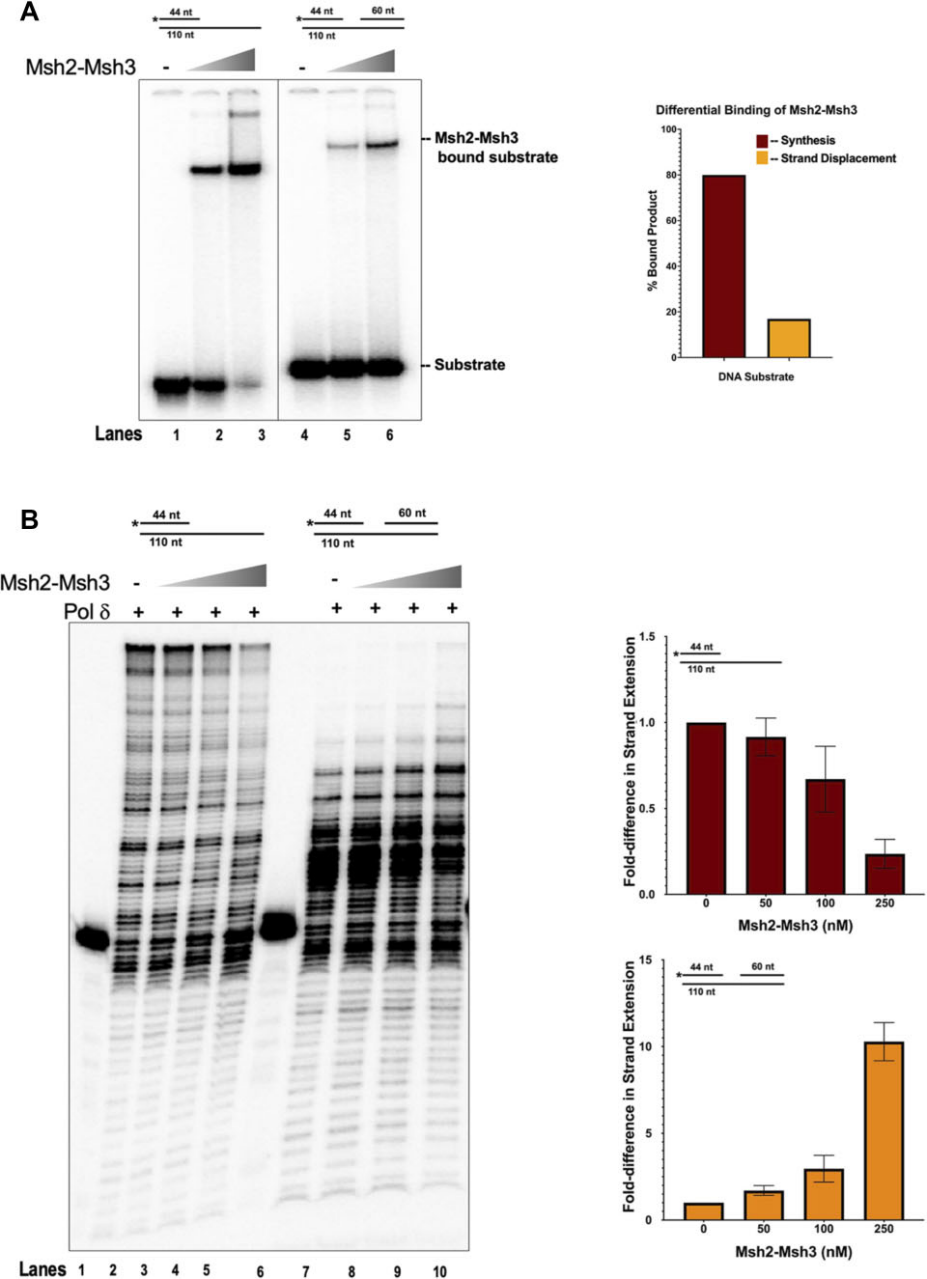
Msh2–Msh3 modulate DNA polymerase δ activity in a DNA substrate-dependent manner. (**A**) Msh2–Msh3 binds to the synthesis substrate (left) and the strand displacement substrate (right) with different affinities. Quantification is shown in right panel. (**B**) Msh2–Msh3 inhibits DNA polymerase δ in the presence of synthesis substrate (left) and stimulates its strand displacement synthesis activity (right).

We previously demonstrated that Msh2–Msh3 interferes with Rad27^FEN1^ and Cdc9^LigI^ activity when allowed to bind the relevant DNA substrates (28). Given that Msh2–Msh3 binds to ss/dsDNA junctions, we considered the possibility that Msh2–Msh3 interacting with different DNA structures might also impact DNA polymerase activity. As Pol δ is likely recruited in both Okazaki fragment processing and LP-BER, we tested Msh2–Msh3’s effect on Pol δ activity *in vitro* in the presence of a simple primer-template (synthesis) DNA substrate or a strand displacement substrate (Figure 12). We compared Msh2–Msh3 substrate binding efficiency on both DNA structures and found Msh2–Msh3 binds efficiently to both, albeit with lower affinity on the strand displacement substrate compared to the synthesis substrate (compare lanes 3 and 6, Figure 12A). Next, we assessed the ability of Msh2–Msh3 to modulate Pol δ synthesis on the synthesis substrate. Titration of Msh2–Msh3 into a primer extension reaction inhibited synthesis by Pol δ (Figure 12A). We observed similar inhibition, albeit to a lesser degree, on the strand displacement substrate [observe substrate retention (44nt) in lanes 8–10, containing Msh2–Msh3] (Figure 12B). However, in contrast to synthesis inhibition, we also observed a stimulation in the strand displacement synthesis products (lanes 8–10). We hypothesize this stimulation to occur on account of Msh2–Msh3 binding to the 5′ primer terminus of the ssDNA/dsDNA junction and transiently opening up the downstream primer allowing for increased Pol δ strand displacement synthesis on a small subset of substrates. These results supported the hypothesis that Msh2–Msh3 can modify DNA metabolism pathways in a DNA structure-dependent manner, with variable impacts on genome stability.

## Discussion

Msh2–Msh3 binds to a wide range of DNA structures (13,14,27,28,68,117,118). In this study, we demonstrated that Msh2–Msh3 could potentially interfere with *in vivo* DNA metabolism pathways that involve distinct DNA structures, including 5′ ssDNA flap intermediates. Even low levels of *MSH3* overexpression increased *in vivo* MMS sensitivity, and higher levels compromised progression through the S phase. This effect was not simply due to Msh2–Msh3 binding to DNA but rather required downstream steps, including ATPase activity and Mlh complexes. *MSH3* overexpression appeared to trigger a *RAD9*-dependent DNA damage checkpoint response and modification of PCNA at K164.

### Maintaining the ‘right’ amount of Msh2-Msh3 is important for genome stability

Altered Msh2–Msh3 expression, up or down, can be deleterious. Downregulation of Msh2–Msh3 is linked to tumorigenesis and cancer (38,47,96), while upregulation of Msh2–Msh3 promotes TNR expansions (93,94). Elevated *Msh2 Msh3* has not been associated with human cancers (96). At the same time, Msh complexes are differentially expressed among organisms and tissues (61,94,96,119,120). For instance, analysis of the abundance of different Msh complexes in actively proliferating murine tissues (including testis, spleen and thymus) showed high expression of Msh6 but no Msh3; on the other hand, low proliferative tissues such as muscle, heart and brain showed high *MSH3* expression levels (119). Msh2–Msh3 protein levels were ∼10× lower than Msh2–Msh6 in human fibroblasts cell lines (121). These differences suggest that proliferating cells produce enough Msh2–Msh3 to participate in processes that maintain genome stability (e.g. MMR and 3′NHTR) but keep Msh3 at lower levels relative to Msh2 and Msh6 to limit aberrant DNA metabolic processes.

We previously demonstrated approximately equal levels of Msh2 and Msh6 levels in logarithmically growing cells, by quantitative immunoblotting (122). While Msh2 and Msh6 expression seem to depend on one another for stability, Msh3 is not stabilized by the presence of either Msh2 or Msh6 (123). Other studies measuring relative levels of Msh2, Msh3 and Msh6 by mass spectrometry (77) or quantitative immuno-purification (76) have observed an excess of Msh2 and Msh6 relative to Msh3. In this study, we assessed the transcript levels of untagged, endogenous *MSH2, MSH6* and *MSH3* by qRT-PCR. Our results indicate that a wild-type yeast strain has a relative endogenous mRNA expression pattern of: Msh6${\mathrm{\ }} \cong {\mathrm{\ }}$Msh2${\mathrm{\ }} >{\mathrm{\ }}$Msh3. Overexpression of Msh3, which generates an imbalance of this distribution, was previously linked with strong mutator phenotypes in human cells (61,62). Consistent with this, when we overexpressed *MSH3* alone, we observed an increase in canavanine resistance, which primarily measures Msh2–Msh6 activity, indicating a decrease in Msh2–Msh6 function (Table 1). Decreased Msh2–Msh6 activity when Msh3 is overexpressed is presumably caused by a reduction in the formation of Msh2–Msh6 protein complexes, disrupting the ‘balance’ between Msh2–Msh6 and Msh2–Msh3 complex formation. When we co-overexpressed *MSH2* and *MSH3* in budding yeast, we observed distinct phenotypes that included sensitivity to alkylating DNA damage (Figure 2), cell cycle delays (Figures 3 and 4) and an enhancement of post-translationally modified PCNA (Figure 5). Our results suggest that controlling the abundance of Msh3 is a mechanism by which cells can limit the interactions of Msh2–Msh3 with DNA structures, including 5′ ssDNA flaps, that modify Msh2–Msh3 function to promote genome instability. A similar correlation between Msh3 levels and TNR expansions was previously observed in a mouse model (94) and human cell lines (93). We predict that both the absolute levels of Msh2–Msh3 and the relative levels of Msh2–Msh3 versus Msh2–Msh6 are important to maintain the ‘right’ balance of MMR activities in different cellular and genomic contexts. Overexpression of *MSH2 MSH6* in yeast disrupted Msh2–Msh6-mediated MMR and heteroduplex rejection, perhaps via sequestration of partner proteins (96). Similarly, overexpression of yeast *MLH1* (124) or *PMS1* (125) disrupted MMR and was predicted to promote formation of non-functional MMR complexes (126).

### An MMR-like response is required for Msh2-Msh3-mediated cell cycle genomic instabilities

Initially, the *in vivo* effects of elevated *MSH3* or *MSH2 MSH3* expression suggested a simple model in which Msh2–Msh3 recognized and bound 5′ ssDNA flap structures as previously demonstrated *in vitro* (14,28), thereby blocking Rad27^FEN1^ activity *in vivo*. This would explain the MMS sensitivity, the defect in cell cycle progression through the S phase and the modification of PCNA. However, two key observations, specifically the requirement for (i) Msh2–Msh3 ATPase activity and (ii) the requirement for *MLH1*, indicated an active, Msh2–Msh3-mediated aberrant MMR-like response reminiscent of current models for Msh2–Msh3’s role in promoting TNR expansions (54,55,65,93,127).

Msh2–Msh3-mediated S phase accumulation and PCNA modification depended on Msh2–Msh3 ATP binding (Walker A) and hydrolysis (Walker B) activity. We and others have previously demonstrated that Msh2–Msh3 DNA binding does not require ATP (13,14,27,72). In fact, the presence of ATP promotes dissociation from DNA *in vitro* (14,27,72,118). Notably, the *msh2* and *msh3* Walker A mutations have dominant-negative effects *in vivo* (63,64); Msh2-msh3G693D inhibited ATP-dependent dissociation from DNA substrates *in vitro* (14), an observation interpreted to be a result of reduced Msh2–Msh3 turnover on the DNA. Therefore, DNA binding alone is not sufficient to produce the adverse effects of *MSH2 MSH3* overexpression. In contrast, *msh3Y925A*, predicted to alter the *regulation* of Msh2–Msh3 ATPase activity, did not reduce Msh2–Msh3-mediated interference with cell cycle progression. *msh3Y925A* also exhibited a dominant negative effect on MMR *in vivo* (64), indicating that this allele interfered with MMR but likely through a distinct mechanism. These observations are consistent with a model in which Msh2–Msh3 is not simply binding to 5′ ssDNA flaps and sterically hindering Rad27^FEN1^-mediated processing, although we predict that this capacity contributes to the cellular phenotypes.

Msh2–Msh3 ATPase activity is required for both Msh2–Msh3-mediated MMR and 3′ NHTR (64,66,128), although there are differential molecular requirements for the regulation of ATPase activity in these two pathways; *msh3Y925A* was defective in MMR but functional in 3′ NHTR (64). Further, Msh2–Msh3 nucleotide binding, hydrolysis and turnover are differentially modulated by MMR versus 3′ NHTR DNA substrates. Msh2–Msh3 ATPase activity is similarly required to promote TNR expansions; mutations in the Msh2 Walker A or Msh3 Walker B motif disrupted TNR expansions in mammalian systems (65,93). Msh2–Msh3 binding to TNR DNA substrates altered nucleotide (ADP and ATP) binding and hydrolysis (27,72,93). We hypothesize that Msh2–Msh3 binding to non-canonical 5′ ssDNA flaps similarly alters Msh2–Msh3’s nucleotide binding/hydrolysis/turnover cycle and initiates an MMR-like response that disrupts OFM. Notably, Msh2–Msh3 may also be directly affecting DNA synthesis by Pol δ in OFM and/or LP-BER. We observed that Msh2–Msh3 either stimulated (synthesis substrate) or inhibited (strand displacement substrate) DNA Pol δ activity *in vitro* in a DNA structure-dependent manner. These observations, coupled with a differential affinity of Msh2–Msh3 for binding these substrates, predict a model in which Msh2–Msh3 binds and alters that conformation of the DNA structures to enhance or inhibit Pol δ activity. *In vivo*, Msh2–Msh3 could, when in sufficient quantities, similarly modulate Pol δ activity, altering and disrupting the kinetics of DNA synthesis.

Loss of Msh2–Msh3 ATPase activity also compromises its recruitment of Mlh complexes. Therefore, the requirement for ATP binding and, to a lesser extent, hydrolysis, may be related to the ability to recruit Mlh complexes. This is consistent with the requirement for *MLH1* to observe the *MSH2 MSH3* overexpression cell cycle phenotype. Loss of *MLH1* eliminates all three Mlh complexes. This suggests a model in which Msh2–Msh3 recruits one or more MLH complexes when bound to 5′ ssDNA flaps and that this interferes with Rad27^FEN1^-mediated pathways. Notably, all three Mlh complexes play a role in Msh2–Msh3-mediated TNR expansion (66,127,129–132). Based on our data, we suggest a model in which Msh2–Msh3 bound to a 5′ ssDNA flap intermediate recruits Mlh complexes, but Msh2–Msh3’s altered ATP binding/hydrolysis activity misregulates Mlh activation, similar to what has been proposed in TNR expansion studies, and interferes with Rad27-mediated OFM.

### A possible role of modified PCNA in Msh2–Msh3-mediated genome instability

The *MSH2 MSH3* overexpression-dependent modification of PCNA at K164 and the abrogation of this phenotype, as well as the cell cycle phenotype, in *elg1Δ* highlights a role for PCNA and its loading/unloading dynamics when the cells responds to elevated Msh2–Msh3 levels. PCNA plays a central role in DNA damage tolerance and repair signaling via post-translational modification. Monoubiquitination of PCNA at K164, catalyzed by the Rad6-Rad18 complex, recruits low-fidelity translesion synthesis polymerases (Pol η, Rev1 and Pol ζ) for potentially mutagenic lesion bypass (105,133–135). PCNA can be further poly-ubiquitinated at K164 by Ubc13-Mms2 and Rad5 to promote high-fidelity recombination (105,136,137). Alternatively, PCNA can be SUMOylated on residues K127 and/or K164, by the Ubc9-Siz1 complex, to prevent recombination through the recruitment of the anti-recombinase Srs2 (105–107,138–140). In this study, we showed that when excess Msh2–Msh3 is present *in vivo*, PCNA post-translational modification at K164 is enhanced (Figure 5, 6). We were unable to detect PCNA ubiquitination by western blot (Supplementary Figure S5) or by mass spectrometry (data not shown). In contrast, the *MSH2 MSH3* overexpression-dependent modification exhibited the same mobility as PCNA following MMS treatment that induces PCNA sumoylation (Supplementary Figure S5B). Further, deletion of *SIZ1*, which is required for PCNA sumoylation, resulted in loss of both the cell cycle phenotype and the PCNA modification (Figure 7). The *siz1Δ* phenotypes and the requirement of *ELG1* for the cell cycle defect to be observed in the context of Msh2–Msh3 expression indicate that the modification is SUMO (Figures 7, 9; Supplementary Figure S5). SUMOylated PCNA is associated with the recruitment of Elg1(110).

The requirement for *ELG1*, which unloads unmodified and sumoylated PCNA to recycle PCNA during lagging strand synthesis (109–111,141), indicated that an alteration in PCNA cycling may be contributing to the *MSH2 MSH3* overexpression-dependent phenotype. Okazaki fragment ligation is required for PCNA recycling by Elg1 (111). We reason that if Msh2–Msh3 is interfering with cell cycle progression by blocking 5′ flap processing, it might also be interrupting the normal cycling of PCNA. Such an interruption could result in the accumulation of SUMOylated PCNA at the replication fork, which would explain the enhancement of modified PCNA observed in Figure 5 upon *MSH2 MSH3* overexpression.

We also note that PCNA interacts with Msh complexes via their PIP-box motifs (142). The PCNA-Msh6 interaction recruits Msh2–Msh6 to the replication fork and plays a critical role in Exo1-independent MMR (19,122). Over-retention of PCNA on the DNA in *elg1Δ* recruits increased Msh2–Msh6, trapping it, accumulating MMR intermediates and resulting in elevated mutation rates and genomic instability (143,144). Excess PCNA might similarly retain and trap excess Msh2–Msh3 in *elg1Δ*, preventing it from interfering with OFM and/or DNA polymerase activity. Excess PCNA may also block Msh2–Msh3 interactions with 5′ flap intermediates, preventing Msh2–Msh3 binding. This could block Msh2–Msh3’s ability to recruit Mlh complexes; work in human Msh2–Msh3 demonstrated overlapping PCNA and MLH interaction motifs such that PCNA and Mlh complexes compete for binding to Msh2–Msh3 (145). As noted above, *MLH1* is required for the *MSH2 MSH3* overexpression phenotypes. (146).

### Model for *MSH2 MSH3* overexpression-mediated genome instability

In this study, we demonstrated that overexpression of the Msh2–Msh3 complex in budding yeast could induce alkylation sensitivity (Figure 2), cell cycle progression delays (Figures 3 and 4) and a putative PCNA-mediated DNA damage response (Figure 8). These phenotypes required functional Msh2–Msh3 ATPase activity (Figures 10 and 11) and were abrogated in *rad9Δ*, *elg1Δ* and *mlh1Δ* backgrounds (Figures 8–11). These data indicate that Msh2–Msh3 has the potential to disrupt many pathways in DNA metabolism, likely through its broad DNA binding capacity (Figure 12). Our findings further support the idea that Msh2–Msh3 binding alone is insufficient to determine between genome stability or instability outcomes. These features are similar to the requirements of Msh2–Msh3 in promoting TNR expansions (27,28,72,147,148), possibly suggesting a common mechanism for promoting genomic instability.

**Figure 13.**
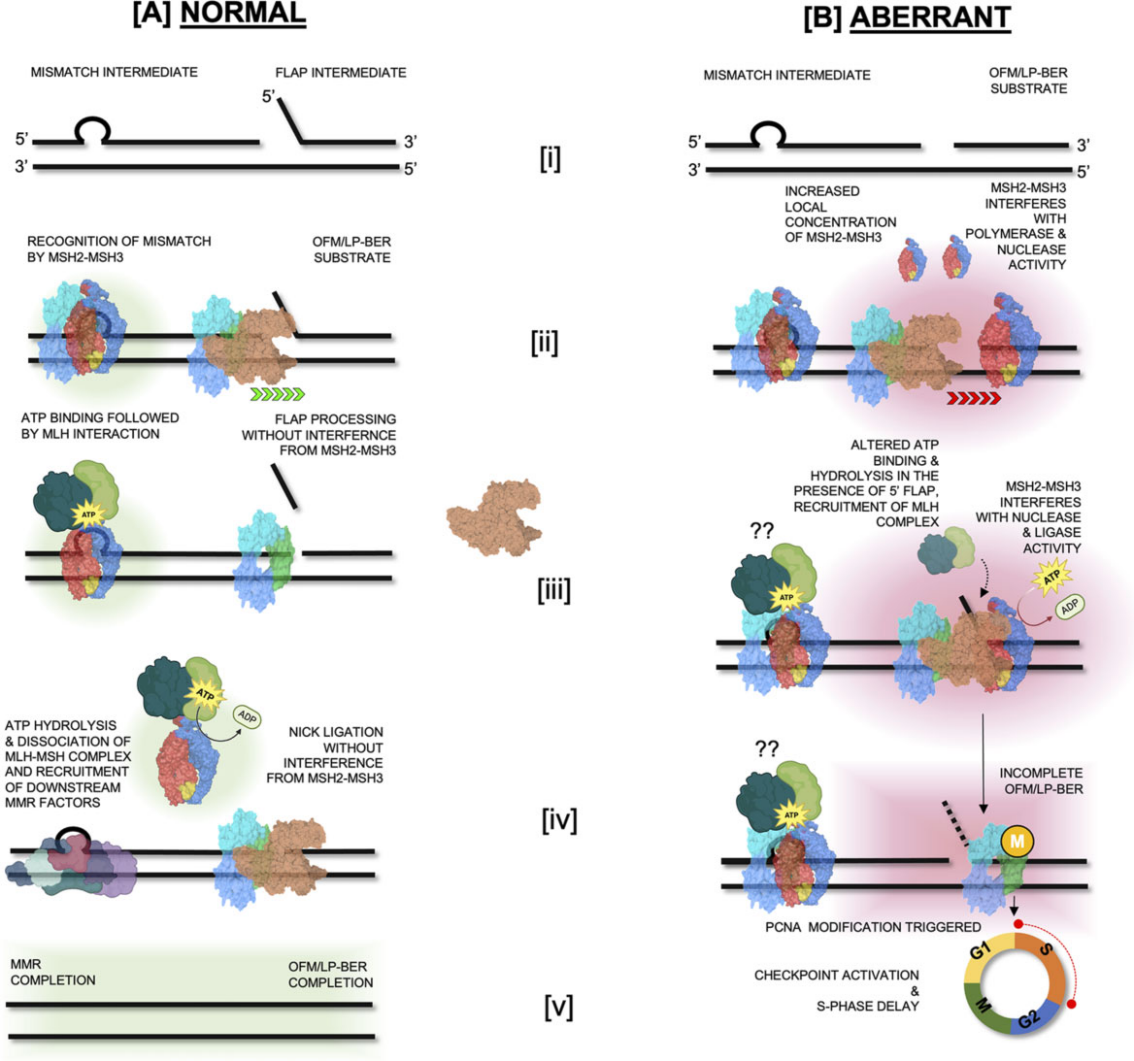
Model of Msh2–Msh3 activity at the replication fork at endogenous and elevated *MSH2 MSH3* expression levels. (**A**) Normal DNA Replication and Repair [i]: Normal structures observed during DNA replication (a mismatch intermediate or flap generated during Okazaki fragment maturation (OFM) or repair (5′ flaps created during long-patch base excision repair (LP-BER); [ii]: Recognition of the mismatch by Msh2–Msh3 and normal Okazaki fragment maturation and 5′ flap synthesis repair during LP-BER–at endogenous protein levels, Msh2–Msh3 is NOT predicted to interact with 5′ flap structures, [iii]: Mismatch binding by Msh2–Msh3 leads to ATP binding and subsequent interaction with Mlh complex. Flaps generated during OFM or LP-BER are cleaved by Rad27*^FEN1^*, [iv]: Msh2–Msh3 hydrolyzes ATP and the MSH-MLH complex dissociates from the mismatch allowing action from the recruited downstream MMR factors. The nick that is generated by flap cleavage is ligated by Cdc9^LigI^ [v]: Successful completion of MMR, OFM and LP-BER. (**B**) Aberrant DNA Replication and Repair on account of elevated Msh2-Msh3 levels [i]: Normal structures observed during DNA replication (a mismatch intermediate or nick generated during OFM or LP-BER, [ii]: increased local concentration of Msh2–Msh3 allows Msh2–Msh3 binding to primer termini and slow down polymerase activity, elevated Msh2–Msh3 may lead to altered MMR loop repair via Msh2–Msh3 oligomerization [iii]: Increased levels Msh2–Msh3 interferes with polymerase synthesis and strand displacement, flap cleavage and nick ligation. Non-canonical DNA structure binding may alter ATP binding and hydrolysis kinetics and possibly recruit MLH complexes. In each scenario, OFM and LP-BER are hindered, MMR may be affected–excess Msh2–Msh3 interferes with Msh2–Msh6 function [iv]: In response to incomplete MMR, OFM and LP-BER, PCNA modification is triggered, [v]: which leads activation of cell cycle checkpoints and delay in cell cycle progression.

We propose the following model for the effects of excess Msh2–Msh3 (Figure 13): (i) Increased Msh2–Msh3 abundance increases its ability to bind 5′ ssDNA flap intermediates during OFM or LP-BER, in addition to MMR loop structures, blocking efficient OFM and potentially activating a *RAD9*-dependent checkpoint response. (ii) Excess Msh2–Msh3 would also bind DNA substrates for Pol δ, either inhibiting (simple primer-template substrate) or enhancing (gapped substrate) its activity and altering the kinetics of DNA synthesis. A direct interaction between Msh2–Msh3 and DNA Pol δ is possible, analogous to a Msh2–Msh3/DNA Pol β interaction in the human system (149). (iii) Binding of a non-canonical DNA structure alters the ATP cycle within the Msh2–Msh3, potentially impacting (promoting?) its interaction with Mlh complexes, as well as turnover of the Msh2–Msh3 complex on the DNA. (*iv*) DNA-bound Msh2–Msh3 recruits Mlh complexes, which in the presence of non-canonical Msh2–Msh3 DNA structures leads to aberrant activation of the Mlh via repeated rounds of nicking in an attempt to repair the DNA, leading to a checkpoint response. Alternatively, Msh2–Msh3 bound to 5′ ssDNA flap structures may misdirect Mlh endonuclease activity to the wrong strand, as demonstrated *in vitro* in the presence of TNR structures (150). The cells responds by slowing progression through the cell cycle, with cells accumulating in S phase, until levels of Msh2–Msh3 are reduced. It is clear that careful regulation of Msh2–Msh3 is critical for preventing aberrant or pathogenic outcomes in DNA metabolism, while retaining its advantageous genome stability functions.

## Supplementary Material

gkad934_supplemental_file

## Data Availability

The data underlying this article are available in the article and in its online supplementary material. Further data underlying this article will be shared on reasonable request to the corresponding author.

## Notes

Present address: Melisa Medina-Rivera, Division of Nutritional Sciences, Cornell University, Ithaca, NY 14850, USA.

Present address: Natalie A. Lamb, Molecular Biology and Strain Development, National Renewable Energy Laboratory, Golden, CO 80401, USA.

Present address: Charanya Kumar, Department of Molecular Biology, Memorial Sloan Kettering Cancer Center, New York, NY 10065, USA.

Present address: Anja Bielinsky, Department of Biochemistry and Molecular Genetics, University of Virginia, Charlottesville, VA 22908, USA.
