# Supplementary material for: Elevated MSH2 MSH3 expression interferes with DNA metabolism in vivo
Source: Nucleic Acids Res. 2023 Nov 1;51(22):12185–206. doi: 10.1093/nar/gkad934 (PMC10711559; doi:10.1093/nar/gkad934)
Supplement: gkad934_supplemental_file [file gkad934_supplemental_file.pdf]

SUPPLEMENTARY TABLES

Table S1 Plasmids used in this study

Plasmid	Allele	Origin; Marker	Reference
pMMR8	*MSH2*	O/E; *TRP1*	(1)
	Constitutive *ADC1* promoter		
pMMR20	*MSH3*	O/E (Gal); *leu2D*	(1)
	Inducible *GAL-PGK* promoter		
pJAS104	Empty Vector	O/E (Gal); *leu2D*	(2)
	Inducible *GAL-PGK* promoter		
pEAE218	*MSH6*	O/E (Gal); *URA3*	(3)
	Inducible *GAL1,10* promoter		
pMME3	*msh3D870A*	O/E (Gal); *leu2D*	This study
	Inducible *GAL-PGK* promoter		
pCK94 or	*msh3Y925A*	O/E (Gal); *leu2D*	(4), This study
pMME2	Inducible *GAL-PGK* promoter		
pCK42	*msh3G796A*	O/E (Gal); *leu2D*	(4)
	Inducible *GAL-PGK* promoter		
pRS424	Empty vector	2μ; *TRP1*	(5)
pSP15	*MSH3*	2μ, *TRP1*	This study
	Endogenous *MSH3* promoter		
pRS425	Empty vector	2μ; *LEU2*	(5)
pSP1	*MSH3*	2μ; *LEU2*	This study
	Endogenous *MSH3* promoter		
pRS414	Empty vector	*ARS CEN; TRP1*	(5)
pSP18	*MSH3*	*ARS CEN; TRP1*	This study
	Endogenous *MSH3* promoter		
pRS415	Empty vector	*ARS CEN; LEU2*	(5)
pSP2	*MSH3*	*ARS CEN; LEU2*	This study
	Endogenous *MSH3* promoter		

Table S2 Strains used in this study

Strain Number	Genotype	Plasmid	Reference
FY23 (S288C background)	*ura3-52, leu2Δ1, trp1Δ63, his3Δ200, lys2Δ202, ura3Δ, leu2Δ, trp1Δ*	none	(6)
JSY905	*msh3Δ* derivative of FY23	none	This study
JSY3971-3972	*msh3Y925A* derivative of FY23	none	(4)
JSY3977-3979	*msh3G796A* derivative of FY23	none	(4)
JSY4418-4420	FY23 *elg1Δ::KanMX*	none	This study
JSY4421-4424	FY23 *mlh1Δ::KanMX*	none	This study
JSY4989-4991	FY23 *rad9Δ::KanMX*	none	This study
JSY5077-5078	FY23 *siz1Δ::KanMX*	none	This study
yb2062 (E133 derived)	*MATα, ade5-1, lys2-A12, trp1-289, his7-2, leu2-3,112, ura3-52, bar1* leu2*::His-POL30 (LEU2), pol30::TRP1*	none	(7)
yb2063 (E133 derived)	*MATα, ade5-1, lys2-A12, trp1-289, his7-2, leu2-3,112, ura3-52, bar1* leu2*::His-pol30K164R (LEU2), pol30::TRP1*	none	(7)
yb2064 (E133 derived)	*MATα, ade5-1, lys2-A12, trp1-289, his7-2, leu2-3,112, ura3-52, bar1* leu2*::His-pol30K242R (LEU2), pol30::TRP1*	none	(7)
yb2066 (E133 derived)	*MATα, ade5-1, lys2-A12, trp1-289, his7-2, leu2-3,112, ura3-52, bar1* leu2*::His-pol30K164R/K242R (LEU2), pol30::TRP1*	none	(7)
JSY4937-45	*yb2062* leu2*::His-POL30 (leu2::hisG), pol30::trp1::hisG*	none	This study
JSY5007-12	*yb2062* leu2*::His-pol30K164R (leu2::hisG), pol30::trp1::hisG*	none	This study
JSY5013-14	*yb2062* leu2*::His-pol30K242R (leu2::hisG), pol30::trp1::hisG*	none	This study
JSY5015-27	*yb2062* leu2*::His-pol30K164R/K242R (leu2::hisG), pol30::trp1::hisG*	none	This study

MSH3-HC* or *MSH3-LC

Strain Number	Genotype	Plasmid	Reference
JSY263	FY23 + LC Empty Vector	pRS415	This study

JSY264	FY23 + *MSH3-LC*	pSP2	This study
JSY265	FY23 + HC Empty Vector	pRS425	This study
JSY265	FY23 + *MSH3-HC*	pSP1	This study
JSY1789-1791 JSY4488-4490	FY23 + LC Empty Vector	pRS414	This study
JSY4183-4185	FY23 + *MSH3-LC*	pSP18	This study
JSY1786-1788	FY23 + HC Empty Vector	pRS424	This study
JSY4180-4182	FY23 + *MSH3-HC*	pSP15	This study
JSY4491-4493	JSY905 + LC Empty Vector	pRS414	This study
JSY4174-4176	JSY905 + *MSH3-LC*	pSP18	This study
JSY4384-4386	JSY905 + HC Empty Vector	pRS424	This study
JSY4171-4173	JSY905 + *MSH3-HC*	pSP15	This study

***MSH2 MSH3* overexpression**

Strain Number	Genotype	Plasmid
JSY1	*ura3-52, trp1, leu2Δ1, his3Δ200, pep4::HIS3, prb1D1.6R, can1, GAL*	
JSY 430	JSY1	pMMR8, pMMR20
JSY4387-4389	JSY1	pMMR8, pMME3
JSY1439-4141	JSY1	pMMR8, pMME2
JSY4136-4138	JSY1	pMMR8, pCK42
JSY4133-4135	JSY1	pMMR8, pMMR20
JSY4130-4132	JSY1	pMMR8, pEAO32
JSY 1505	*msh3Δ* derivative of JSY1	
JSY 2329-2331	JSY1505	pMMR8, pCK42
JSY 2520-2522	JSY1505	pMMR8, pCK94
JSY4402-4404	JSY1505	pMMR8, pCK42
JSY4399-4401	JSY1505	pMMR8, pMMR20
JSY4396-4398	JSY1505	pMMR8, pMME3
JSY4166-4168	JSY1505	pMMR8, pMME2
JSY321-323	FY23	pRS424, pMMR20
JSY4390-4392	FY23	pMMR8, pMME3
JSY4151-4153	FY23	pMMR8, pMME2
JSY4148-4150	FY23	pMMR8, pCK42
JSY4145-4147	FY23	pMMR8, pMMR20

JSY4142-4144	FY23	pMMR8, pRS425
JSY4393-4395	JSY905	pMMR8, pMME3
JSY4163-4165	JSY905	pMMR8, pMME2
JSY4160-4162	JSY905	pMMR8, pCK42
JSY4157-4159	JSY905	pMMR8, pMMR20
JSY4153-4156	JSY905	pMMR8, pRS425
JSY4444-4446	JSY4418	pMMR8, pMMR20
JSY4992-4997	JSY4989, 4990	pMMR8, pMMR20
JSY4447-4449	JSY4421	pMMR8, pMMR20
JSY5082-5085	JSY5077	pMMR8, pMMR20
JSY5079-5081	JSY4942	pMMR8, pMMR20
JSY5028-5033	JSY5010, 5011	pMMR8, pMMR20
JSY5034-5039	JSY5013, 5014	pMMR8, pMMR20
JSY5040-5045	JSY5015	pMMR8, pMMR20

Table S3 Oligonucleotides used for qRT-PCR

Oligo	Gene	Sequence (5'–3')	Product size
SO 351	*MSH2*	GACAAGCAACAATCGGCTCTGGTT	172 bp
SO 352		TCCATGGGATGCAACTTGGGTCTA	
SO 355	*MSH3*	TGCGTACTGTTCTTTCCCGGATGT	177 bp
SO 356		CTTGATTTGCTGGCACCTGGATCA	
SO 317	*MSH6*	TACCTTCTGGCACACCGTCAAAGA	107 bp
SO 318		TGCCTGTCTTTCCTCCTTGTGGAT	
SO 319	*PDA1*	AACGCCAACCATCACAATTGGTCC	173 bp
SO 320		ACGACTCGAAGGAAGATTCAGGCA	

Table S4: Oligonucleotides used for *in vitro* Polymerase δ assays

Primer	Length	Sequence

T1	110	**3’**CAGGTGGGCGCGGTGGAGGACGAAGTTACACGACCTAGGATGTTGTTCTGCTTAAGCCTATGCTCCGGTCACGGCTGCACGGTCGGATTAAAGTTAGGTGG**5’**
U1	44	5’ GTCCACCCGCGCCACCTCCTGCTTCAATGTGCTGGATCCTA3’
D1	60	5’CAGGTGGGCGCGGTGGAGGCCGTCACGGCTGGCAGGTCGGATTTAAGTTAGGTGGG3’

SUPPLEMENTARY REFERENCES

1. Habraken, Y., Sung, P., Prakash, L. and Prakash, S. (1996) Binding of insertion/deletion DNA mismatches by the heterodimer of yeast mismatch repair proteins MSH2 and MSH3. *Curr Biol*, **6**, 1185-1187.
2. Eichmiller, R., Medina-Rivera, M., DeSanto, R., Minca, E., Kim, C., Holland, C., Seol, J.H., Schmit, M., Oramus, D., Smith, J. *et al.* (2018) Coordination of Rad1-Rad10 interactions with Msh2-Msh3, Saw1 and RPA is essential for functional 3' non-homologous tail removal. *Nucleic Acids Res*, **46**, 5075-5096.
3. Kijas, A.W., Studamire, B. and Alani, E. (2003) Msh2 separation of function mutations confer defects in the initiation steps of mismatch repair. *J Mol Biol*, **331**, 123-138.
4. Kumar, C., Williams, G.M., Havens, B., Dinicola, M.K. and Surtees, J.A. (2013) Distinct requirements within the Msh3 nucleotide binding pocket for mismatch and double-strand break repair. *J Mol Biol*, **425**, 1881-1898.
5. Sikorski, R.S. and Hieter, P. (1989) A system of shuttle vectors and yeast host strains designed for efficient manipulation of DNA in Saccharomyces cerevisiae. *Genetics*, **122**, 19-27.
6. Madison, J.M. and Winston, F. (1997) Evidence that Spt3 functionally interacts with Mot1, TFIIA, and TATA-binding protein to confer promoter-specific transcriptional control in Saccharomyces cerevisiae. *Mol Cell Biol*, **17**, 287-295.
7. Becker, J.R., Gallo, D., Leung, W., Croissant, T., Thu, Y.M., Nguyen, H.D., Starr, T.K., Brown, G.W. and Bielinsky, A.K. (2018) Flap endonuclease overexpression drives genome instability and DNA damage hypersensitivity in a PCNA-dependent manner. *Nucleic Acids Res*, **46**, 5634-5650.

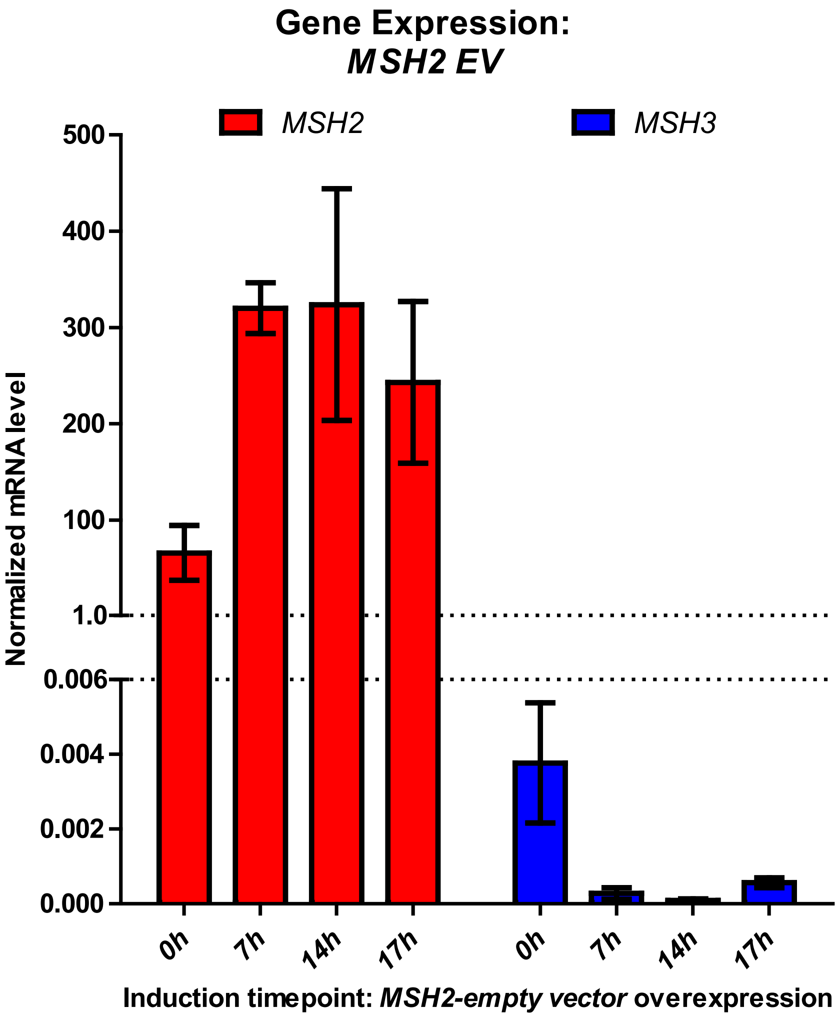

Figure S1. *MSH2* + EV control shows no *msh3* mRNA.

RNA was isolated from strains overexpressing *MSH2* under a constitutive promoter along with an empty vector plasmid (pJAS104). Endogenous mRNA levels of *MSH2* (pink, left) and *MSH3* (blue, right) were measured using qRT-PCR. All RNA levels were normalized to the reference gene *PDA1*. Bars represent mean ± SEM.

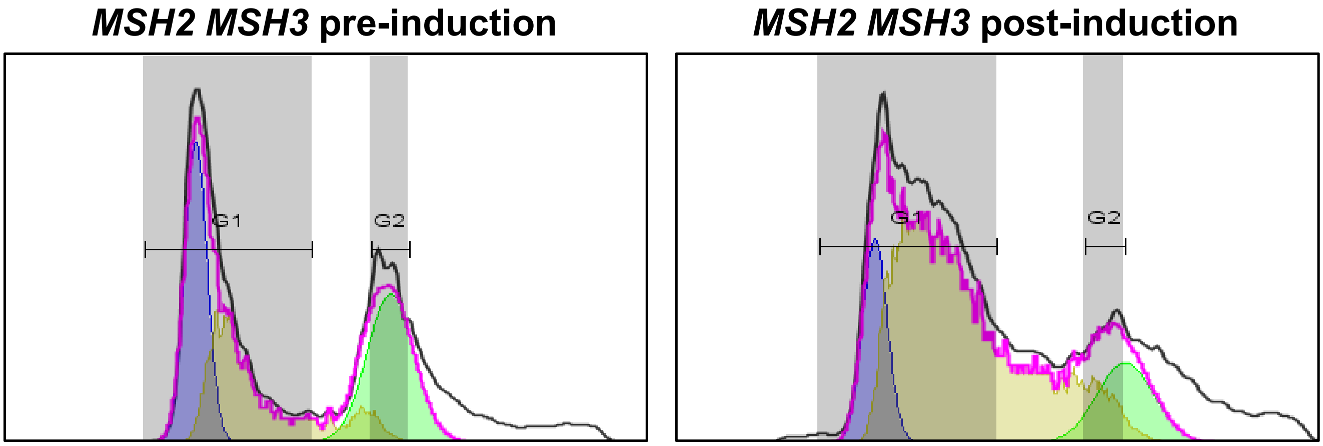

Figure S2. Representation of BD FlowJo™ Quantification of Flow Cytometry data.

Graphical representation of each time point accounts for a gated subset of the ten thousand total events recorded during each flow cytometry analysis. Gates P1, P2, and P3 excluded cellular debris and doublet events. Data shown on histogram in black represents cells in gate P3. Cell cycle analysis functionality inherent to the software was applied to P3 populations. Cells in G1, S, and G2/M are represented by areas under the blue (left), yellow (center), and green (right) curves, respectively. The pink line represents the sum of the curves. Ranges constraining the G1 and G2 peaks (vertical, gray) were tailored by sample.

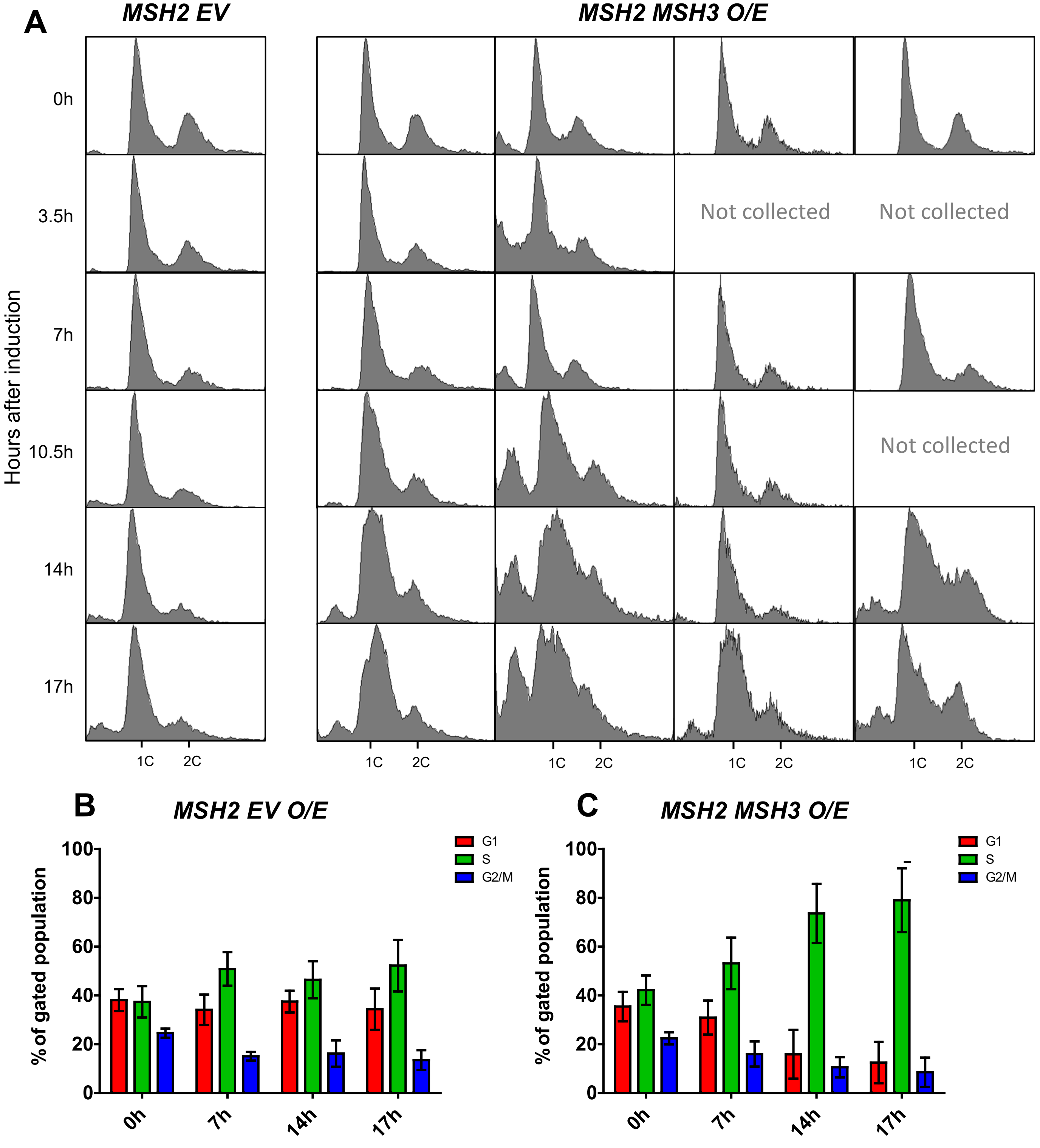

Figure S3. *MSH2 MSH3* overexpression induces a delay in cell cycle progression.
MSH2 and *MSH3* were overexpressed following galactose induction in a *msh3Δ* background. Aliquots were collected at indicated hours after induction. (A) Histograms are shown of chromosomal content of asynchronous populations of *MSH2* + *empty vector (EV)* and *MSH2* + *MSH3* at several timepoints following addition of galactose. Flow cytometry experiments were repeated at least three times, with at least two independent transformants. 1C indicates 1x DNA content; 2C indicates 2x DNA content. (B and C) Quantification of relative proportion of cells in different phases of the cell cycle for *MSH2* + *EV* (B) or *MSH2* + *MSH3* (C). The percentage of cells in G1 (1C), G2/M (2C) or S (between 1C and 2C) phases was determined using FlowJo software (see Figure S1 for details). Plotted values correspond to data collected from at least three independent experiments. Error bars represent SEM.

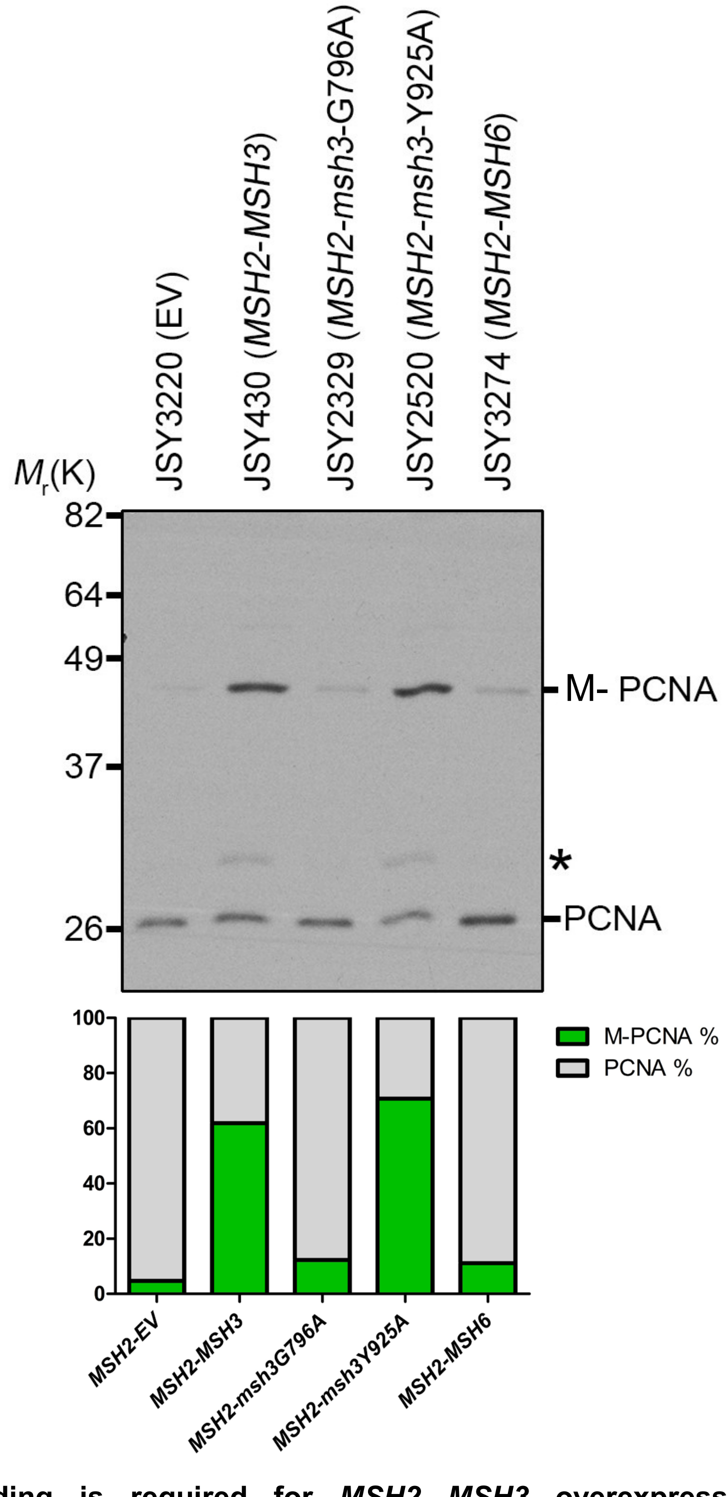

Figure S4. ATP binding is required for *MSH2 MSH3* overexpression-induced PCNA modification, but proper regulation of ATP binding is not.

MSH2 and *MSH3* or *msh3* alleles were overexpressed in a *msh3Δ* background, as previously described. Samples were taken following induction and TCA protein extracts were prepared and the proteins were separated by 10% SDS-PAGE, transferred into a membrane and then probed with anti-PCNA. Modified PCNA is marked as M-PCNA. The asterisk indicates non-specific bands. Quantification of bands was done using ImageJ software and shown on the below graph as relative percentages of the total PCNA signal in that late on the blot shown (sum of modified & unmodified bands). Pairwise t-tests indicated that the proportion of M-PCNA in *MSH2 MSH3 O/E* was statistically different from that in *MSH2 msh3G796A* ($p<0.0001$) and *MSH2 MSH6 O/E* ($p=0.0009$), but not *MSH2 msh3Y925A* ($p=0.6901$).

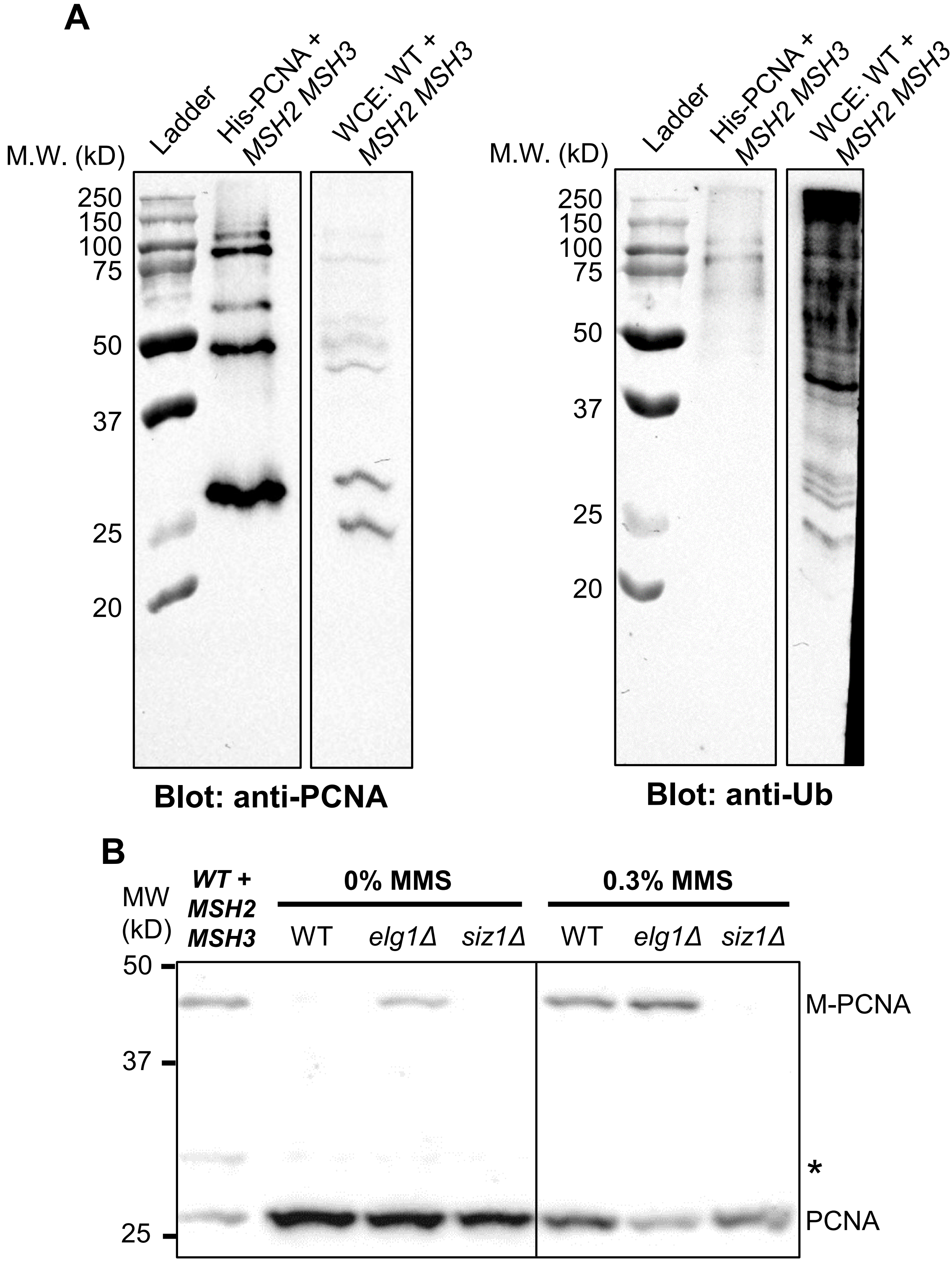

Figure S5. Modified PCNA band may be SUMO-PCNA.
(A) *MSH2* and *MSH3* were overexpressed as described in a strain carrying polyhistidine-tagged, wild-type PCNA *(His-POL30)*. Following induction, His-tagged PCNA was enriched using Ni-NTA agarose beads. Enriched His-PCNA samples ("His-PCNA + 2-3") were analyzed by Western blot. Following *MSH2 MSH3* induction in wild-type strain, whole-cell extracts (WCE) were collected by TCA precipitation as described and analyzed by Western blot ("WCE: WT 2-3"). Samples were immunoblotted for PCNA (left) or for ubiquitin (right). **(B)** Wild-type, *elg1Δ*, and *siz1Δ* cells were treated with 0% or 0.3% MMS in mid-log phase for 90 minutes. Whole-cell extracts were collected by TCA precipitation and analyzed by Western blot for PCNA as described.

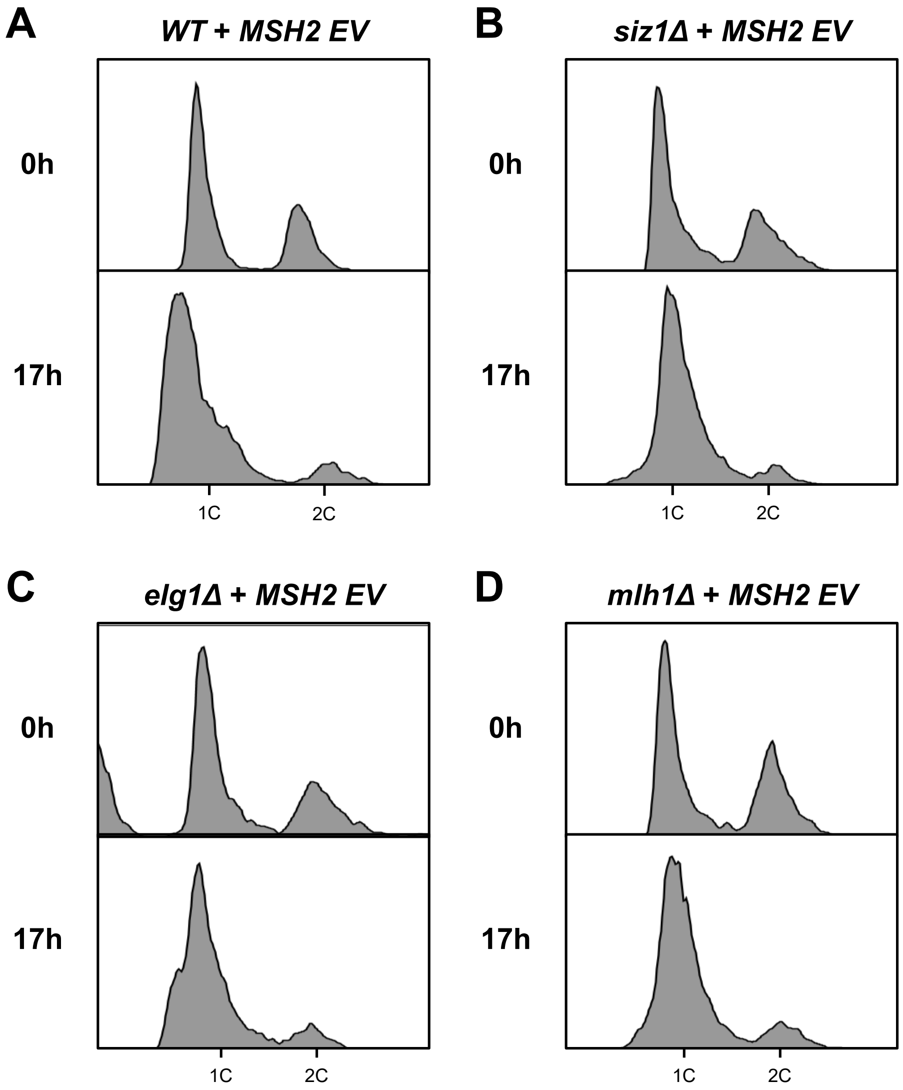

Figure S6. Overexpression of *MSH2 EV* does not cause S phase accumulation in deletion strains.
Representative hisotgrams of *MSH2* and empty vector (EV) overexpressed in S288C wild-type **(A)**, *siz1Δ* **(B)**, *elg1Δ* **(C)**, or *mlh1Δ* **(D)** backgrounds and induced as previously described. Cells were harvested for flow cytometry before and after induction.

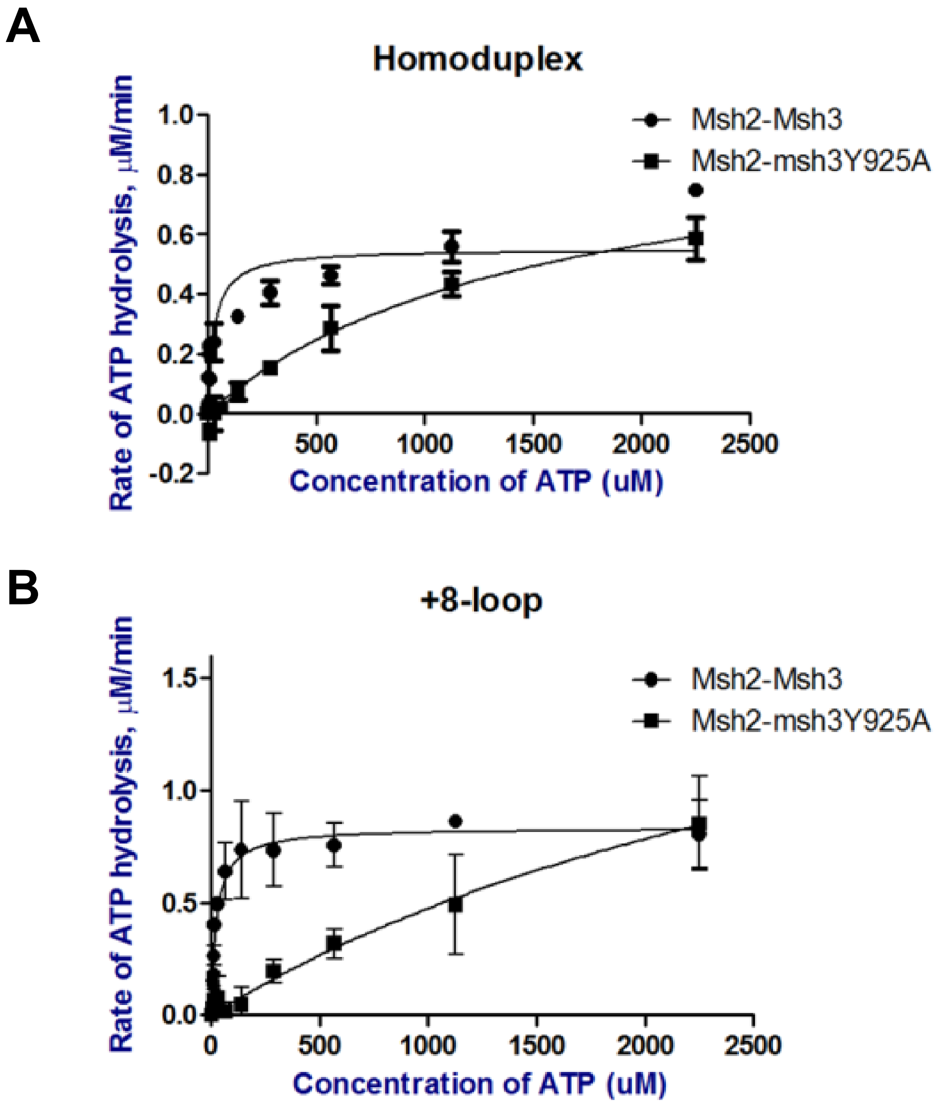

Figure S7. ATPase activity of Msh2-Msh3 and Msh2-msh3Y925A in the presence of homoduplex and +8 loop (MMR) DNA substrates.

The rate of hydrolysis was plotted against the concentration of ATP for wild-type Msh2-Msh3 (circles) and Msh2-msh3Y925A (squares). ATP hydrolysis was measured in the presenc of non-speciific homoduplex (**A**) or specific +8 loop (**B**) DNA substrates.
